# Efficacy and safety of micafungin versus extensive azoles in the prevention and treatment of invasive fungal infections for neutropenia patients with hematological malignancies: A meta-analysis of randomized controlled trials

**DOI:** 10.1371/journal.pone.0180050

**Published:** 2017-07-12

**Authors:** Cho-Hao Lee, Jung-Chung Lin, Ching-Liang Ho, Min Sun, Wel-Ting Yen, Chin Lin

**Affiliations:** 1 Department of Hematology and Oncology Medicine, Tri-Service General Hospital, National Defense Medical Center, Taipei, Taiwan, ROC; 2 Department of Internal Medicine, Tri-Service General Hospital, National Defense Medical Center, Taipei, Taiwan, ROC; 3 Department of Infectious Diseases and Tropical Medicine, Tri-Service General Hospital, National Defense Medical Center, Taipei, Taiwan, ROC; 4 Department of General Surgery, Taihe Hospital, Hubei University of Medicine, Shiyan, P.R. China; 5 School of Medicine, National Defense Medical Center, Taipei, Taiwan, ROC; 6 School of Public Health, National Defense Medical Center, Taipei, Taiwan, ROC; 7 Department of Research and Development, National Defense Medical Center, Taipei, Taiwan, ROC; Yonsei University, REPUBLIC OF KOREA

## Abstract

**Background:**

Current studies that compare the efficacy and safety of micafungin (MCFG) with that of triazoles for the prophylaxis and treatment of invasive fungal infections (IFIs) demonstrate a lack of sufficient evidence and yield conflicting results. To compare the efficacy and safety of MCFG and triazoles in the prevention and treatment of IFIs, we conducted a meta-analysis and trial sequential analysis (TSA).

**Methods:**

For the meta-analysis, we systematically searched the databases of PubMed, Embase and Cochrane Central Register of Controlled Trials and relevant database articles for randomized controlled studies published through November 2016. Comparative studies of the efficacy and safety of MCFG versus triazoles in the prevention and treatment of IFIs were selected. Meta-analysis was performed by R software with the “metafor” package. Pooled results were expressed as risk ratios (RRs) with corresponding 95% confidence intervals (CI). TSA was adopted to assess the studies’ power with TSA version 0.9 beta.

**Results:**

Nine current studies were included in the meta-analysis (1049 cases and 959 controls). Pooled trial comparisons indicated that MCFG does have significantly higher treatment success rates (RR = 1.13; 95% CI, 1.02–1.25; p = 0.0205) and reduces the number of overall IFIs (RR = 0.75; 95% CI, 0.61–0.92; p = 0.0056). However, MCFG demonstrates no difference in all-cause mortality (RR = 0.76; 95% CI, 0.52–1.12, p = 0.1624). For the safety evaluation, MCFG had a significantly lower incidence of severe adverse events (AEs) (RR = 0.45; 95% CI, 0.25–0.83; p = 0.0105), hepatic impairment (RR = 0.70; 95% CI, 0.50–0.97; p = 0.0363) and premature discontinuation (RR = 0.51; 95% CI, 0.34–0.76, p = 0.0010). Meta-regression analysis disclosed the correction of mean age and treatment success rates (P < 0.0001). Meanwhile, TSA demonstrated sufficient power to show efficacy.

**Conclusions:**

The treatment success rate of MCFG is superior to that of triazoles for the prophylaxis and treatment of IFIs, and correction of the mean patient age demonstrates that efficacy increases as patient age decreases. MCFG appears to be well-tolerated with manageable side effects and lower withdrawal rates. However, additional clinical trials should be conducted on specific drug-related mortality and AEs to gather sufficient evidence on these matters.

## Introduction

Invasive fungal infections (IFIs) are a significant cause of morbidity and mortality for neutropenia patients. The risk of IFIs is particularly increased in patients with hematologic malignancy undergoing intensive chemotherapy or stem cell transplantation [1, 2]. Furthermore, invasive mold infections often occur exclusively in high-risk patients with profound neutropenia (<100 cells/mm3) lasting longer than 10–15 days [3–5]. Now that the threats posed by bacterial and viral infections are somewhat reduced, IFIs have become one of the main infective causes of mortality in this population [6].

Over the past 20 years, a series of studies have assessed the effect of anti-fungal agents for the prophylactic therapy of IFIs. Although numerous anti-fungals are available, IFIs remain a serious problem because of obstacles to timely diagnosis and high morbidity and mortality rates associated with such infections. Recent randomized trials [7–10] and meta-analyses [11–13] showed a reduced risk of IFIs in patients who used triazoles such as fluconazole (FLCZ) and itraconazole (ITCZ) for invasive candidemia (IC), and voriconazole (VOCZ) for invasive aspergillosis (IA).

MCFG was first introduced in Japan in 2002. It is a new member of the echinocandin class of anti-fungals that are effective against both IC and IA, and preliminary clinical studies have shown that it demonstrates good anti-fungal activity [14, 15]. The US Food and Drug Administration (FDA) approved MCFG for anti-fungal prophylaxis during the pre-engraftment phase in patients undergoing hematopoietic stem cell transplantation (SCT). The Infectious Diseases Society of America (IDSA) guidelines also recommend MCFG as an alternative prophylactic drug to treat IA [16].

Previous systematic reviews and meta-analyses demonstrated that MCFG was more effective in the reduction of IFIs than FLCZ and ITCZ as a prophylactic therapy and as a treatment for neutropenia patients [12]. However, XU, S.X., 2016 only conducted trials until Dec. 2013 and combined randomized controlled trials and cohort studies [17]. Wang, J.F., 2015 compared different echinocandins with different triazoles in different immunocompromized patients with diseases such as HIV, kidney transplantation and chronic pulmonary aspergillosis [12]. Recently published clinical reports provide additional information regarding the efficacy and safety of MCFG in febrile neutropenia (FN) patients [7–10].

The aim of this study was to use data from randomized controlled studies to compare the clinical efficacy and safety of MCFG in the prevention and treatment of IFIs to that of extensive azole anti-fungal agents in neutropenia patients with hematological malignancy during intensive chemotherapy or SCT.

## Materials and methods

### 2.1 Search strategy

A comprehensive literature search was performed to identify studies published through November 2016. The main sources comprised of the PubMed, EMBASE, Cochrane Library, and Clinical Trials databases. Electronic searches were performed with a combination of MeSH terms, Emtree synonyms and free words using the following search algorithm. Other routes (e.g., hand search and library resource sharing) were also considered. The search terms comprised of micafungin, micafungin sodium, micamine, FK 463, Echinocandin, Lipopeptides, anti-fungal agents, FN, and neutropenic fever. The publication language of studies was not limited. The specific searching strategy is described in S1 Table.

### 2.2 Inclusion and exclusion criteria

The PRISMA checklist is described in S2 Table. This study focused on the hematologic malignancy population during FN and aimed to compare the efficacy and safety of MCFG to that of extensive azole anti-fungal agents. All articles published from the dates of inception of these medical databases to November 2016 were included.

All related studies that compared the use of MCFG to the use of triazoles in neutropenic fever were considered for inclusion. The inclusion criteria were as follows: (1) randomized controlled studies, (2) compared efficacy or incidence of AEs in 2 comparable populations, (3) received intravenous MCFG for anti-fungal prevention or treatment and (3) FN defined as absolute neutrophil count < 1500/uL. If the published data was incomplete, we made attempts to contact the authors for further information. Studies were excluded if they: (1) were incomplete or included duplicated data, (2) did not contain any predetermined clinical outcomes, or (3) could not be pooled with other included studies.

### 2.3 Data extraction

2 reviewers (CHL and CL) independently extracted data and assessed the risk of bias. For each article, we recorded the first author’s name, year of publication, study design, region, definition of the intervention group, population characteristics (mean age, acute leukemia percent, previous therapy including chemotherapy or hematopoietic stem cell transplantation and allogenic SCT percent), therapeutic regimen (drug name, dose and administration routine), outcome measures and study results. When more than one publication about the same study existed, only the publication with the most complete data was included in the analysis. Available data was demonstrated on S4 Table.

### 2.4 Quality assessment

The quality of the included articles was assessed by 2 authors (CHL and CL) according to the Cochrane Collaboration Reviewers’ Handbook for Systematic Reviews of Interventions [18]. Trials scored 1 point for each area addressed in the study design (randomization, concealment of allocation, blinding, reporting of withdrawals, selective reporting and other bias), with a possible score ranging from 0 (lowest level of quality) to 7 (highest level of quality) [19]. If the authors disagreed on the assessment of any of these points, they reached a consensus through discussion of the matter. The quality assessment of the included studies is described in S3 Table.

### 2.5 Statistical analysis

The population characteristics of each included study are presented as means or proportions where appropriate. Our meta-analysis examined the efficacy and safety of MCFG as compared to that of triazoles in FN patients in each study using RRs with 95% CIs. Egger’s regression and a funnel plot were used to test the publication bias of pooled results [20]. I^2^ was calculated with the Cochrane Q test and used to quantify heterogeneity; an I^2^ value >40% indicated a moderate to high heterogeneity [21].

The source of heterogeneity was explored with a meta-regression using an average summary value. Possible moderators (age, quality, acute leukemia percent, transplant type, neutropenic duration, study duration and previous chemotherapy or SCT) were tested to explore heterogeneity. This study considered a p-value of <0.05 to be significant for all analyses. Statistical analyses were conducted using the “metafor” [22] and “meta” [23] packages of R software, version 3.2.3.

Trial sequential analysis (TSA) was conducted [24, 25], which is similar to interim analyses in a single trial. Monitoring boundaries are used to decide whether a trial could be terminated early when a p-value is sufficiently small to show the anticipated effect. We used monitoring boundaries to determine if a study produced a sufficiently small p-value to demonstrate the anticipated power. Random errors may be increased due to insufficient comparisons and repetitive testing of pooling data when the estimated information samples have not been achieved [26–28]. TSA version 0.9 beta (www.ctu.dk/tsa) was used for quantification of the needed information samples.

## Results

### 3.1 Characteristics of studies

**Fig 1:**
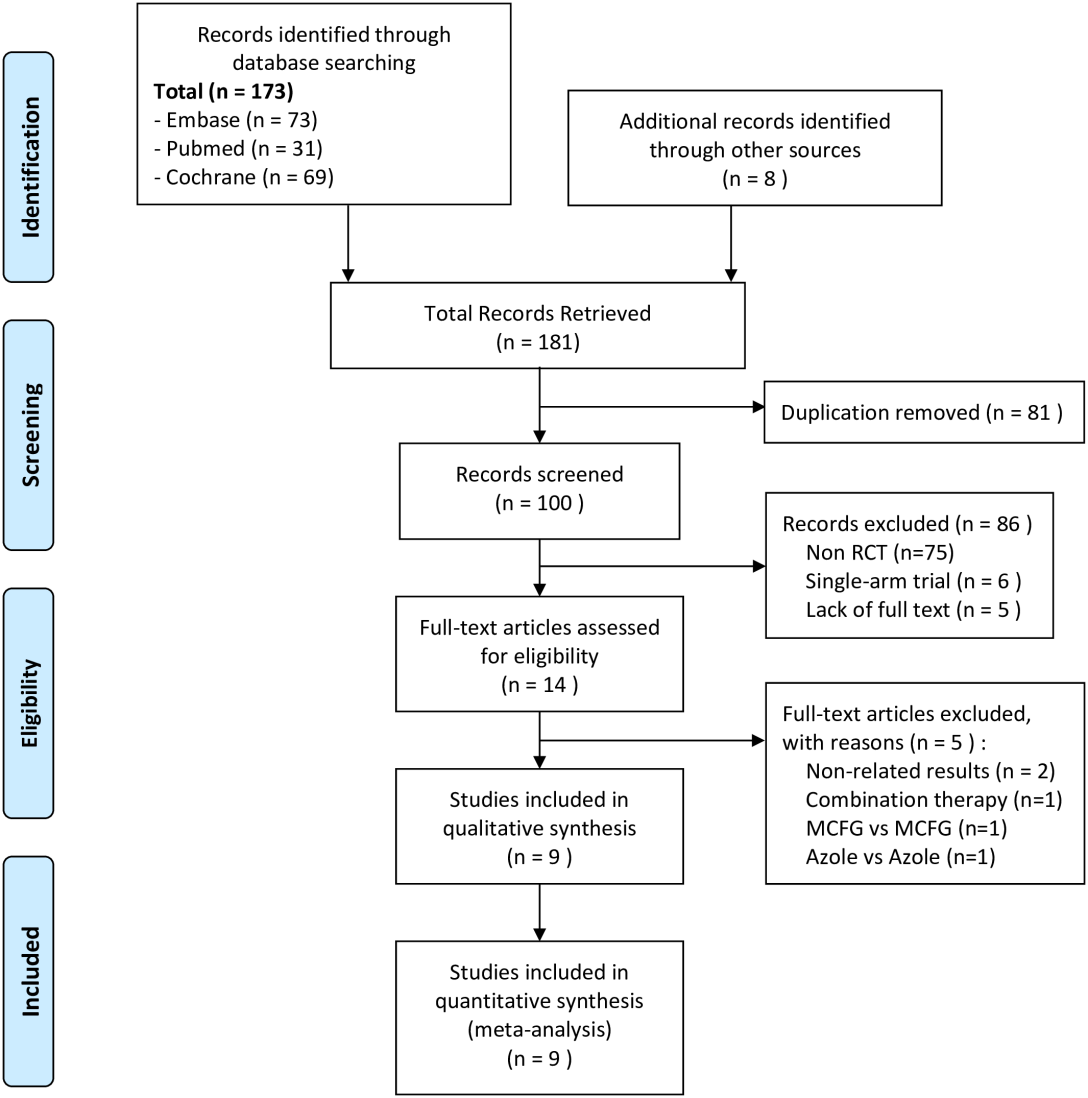
Flow diagram of the identification process for eligible studies.

Fig 1 discloses the comprehensive literature search results. The initial search yielded 181 articles. Of these 181 results, 9 articles that described randomized controlled trials (RCTs) involving 2008 patients were ultimately included in the present meta-analysis [7–10, 14, 29–32]. The detailed selection process is presented in S1 Table, and characteristics of the included studies are described in Table 1. 7 studies are for prophylaxis and 2 are for empiric treatment. 5 studies are multicenter trials, 2 are double-blind trials, 4 are open-label design and 2 are unclear design. 5 studies assessed the drug safety and efficacy comparison with FLCZ, 2 with ITCZ and 2 with VOCZ. Patient mean age ranged from 6.01 to 53.0 years and the follow up time was from 28 days to 6 months. The trials were conducted in different countries. 2 trials were conducted in the United States, 2 in Japan, 3 in Korea, 1 in China and 1 in Egypt (Table 1).

**Table 1:** Basic characteristics of included randomized trials.

Author Year	Trial Design	Country	Cases (N)	Mean Age	Main Therapy	MCFG Dosage	Control Azole-Drug	Purpose	Definition of Treatment Success
Burik et al. 2004 (14)	MC,DB,RCT	US	882 (425 MCFG; 457 FLCZ)	42.5	SCT	1mg/kg, once/day	FLCZ, 8mg/kg, once/day	Prophylaxis	Absence of proven, probable, or suspected systemic fungal infection through the end of prophylaxis therapy Absence of a proven or probable systemic fungal infection through the end of the 4-week post-treatment period.
Hiemenz et al. 2005 (29)	DB,RCT	US	74 (62 MCFG; 12 FLCZ)	43.2	SCT	12.5,25,50, 75,100,150,mg/day	FLCZ, 400mg, once/day	Prophylaxis	Incidence of treatment-emergent fungal infections during treatment and post-treatment Requirement for empirical antifungal therapy.
Hiramatsu et al. 2008 (30)	MC,OP,RCT	Japan	104 (52 MCFG; 52 FLCZ)	46.9	SCT	150mg,once/day	FLCZ, 400mg, once/day	Prophylaxis	Absence of proven, probable, or suspected systemic fungal infection through the end of prophylaxis therapy Absence of a proven or probable systemic fungal infection through the end of the 4-week post-treatment period.
Sawada et al. 2009 (31)	MC,RCT	Japan	107 (54 MCFG; 53 FLCZ)	6.01	Both	2mg/kg,once/day	FLCZ, 10mg,kg, once/day	Prophylaxis	Absence of proven, probable, or suspected systemic fungal infection through the end of prophylaxis therapy Absence of a proven or probable systemic fungal infection through the end of the 10-days post-treatment period.
Huang et al. 2012 (32)	MC,OP,RCT	China	287 (140 MCFG; 147 ITCZ)	32.7	SCT	50mg,once/day	ITCZ, 5mg/kg, once/day	Prophylaxis	Absence of proven, probable, or suspected systemic fungal infection through the end of prophylactic therapy Absence of a proven or probable systemic fungal infection through the end of the 4-week post-treatment period.
Oyake et al. 2015 (9)	OP, RCT	Japan	100 (50 MCFG; 50 VOCZ)	53.0	C/T	150mg,once/day	VOCZ, 4mg/kg, twice/day	Empiric	Successful treatment of basal fungal infection Absence of breakthrough fungal infection Survival for ≥ 7 days after completion study therapy Study therapy discontinued prematurely because of toxicity or lack of efficacy Resolution of fever during neutropenia
Jeong et al. 2016 (8)	MC, RCT	Korean	153 (77 MCFG; 76 ITCZ)	49.0	C/T	100mg,once/day	ITCZ, 200mg, twice/day	Empiric	Not have breakthrough invasive fungal infection (IFI) Survived for 7 days after therapy ended No premature discontinuation because of adverse events or lack of efficacy Defervescence during granulocytic nadir Successful treatment of any baseline fungal infection
Park et al. 2016 (34)	OP, RCT	Korean	257 (168 MCFG; 89 FLCZ)	46.7	SCT	50mg,once/day	FLCZ, 400mg, once/day	Prophylaxis	No incidence of proven or probable IFIs during the 100 days after HSCT.
Mahmoud et al.* 2016 (7)	CC, RCT	Egypt	70 (35 MCFG; 35 VOCZ)	7.35	C/T	50mg,once/day	VOCZ, 4mg/kg, twice/day	Prophylaxis	No development of proven, probable, or possible fungal infection according to revised definitions of EORTC/MSG consensus group

C/T: Chemotherapy SCT: Stem cell transplant MC: Multi-center DB: Double blind OP: Open label CC: Case control

*: a MSc degree study which was unpublished yet.

### 3.2 Quality of the individual study

Risk of bias evaluation is described in S1A Fig. 4 studies employed the appropriate random sequence generation and allocation concealment [8, 9, 14, 32], while others were unclear due to their failure to state the detailed randomization and allocation method [10, 29, 30]. Authors of 2 RCTs reported that they employed blinded assessors [14, 29] while others were open-label or unclear trials. The risk of bias for reporting participant dropout or withdrawal was low in 8 RCTs, while the remaining trials had a high risk of reporting bias [10]. Additional findings regarding the risk of bias are summarized in S1B Fig (a detailed description is presented in S3 Table). All studies that were deemed to be of adequate quality were included in data synthesis.

### 3.3 Efficacy outcome

#### 3.3.1 Treatment success rate

**Fig 2:**
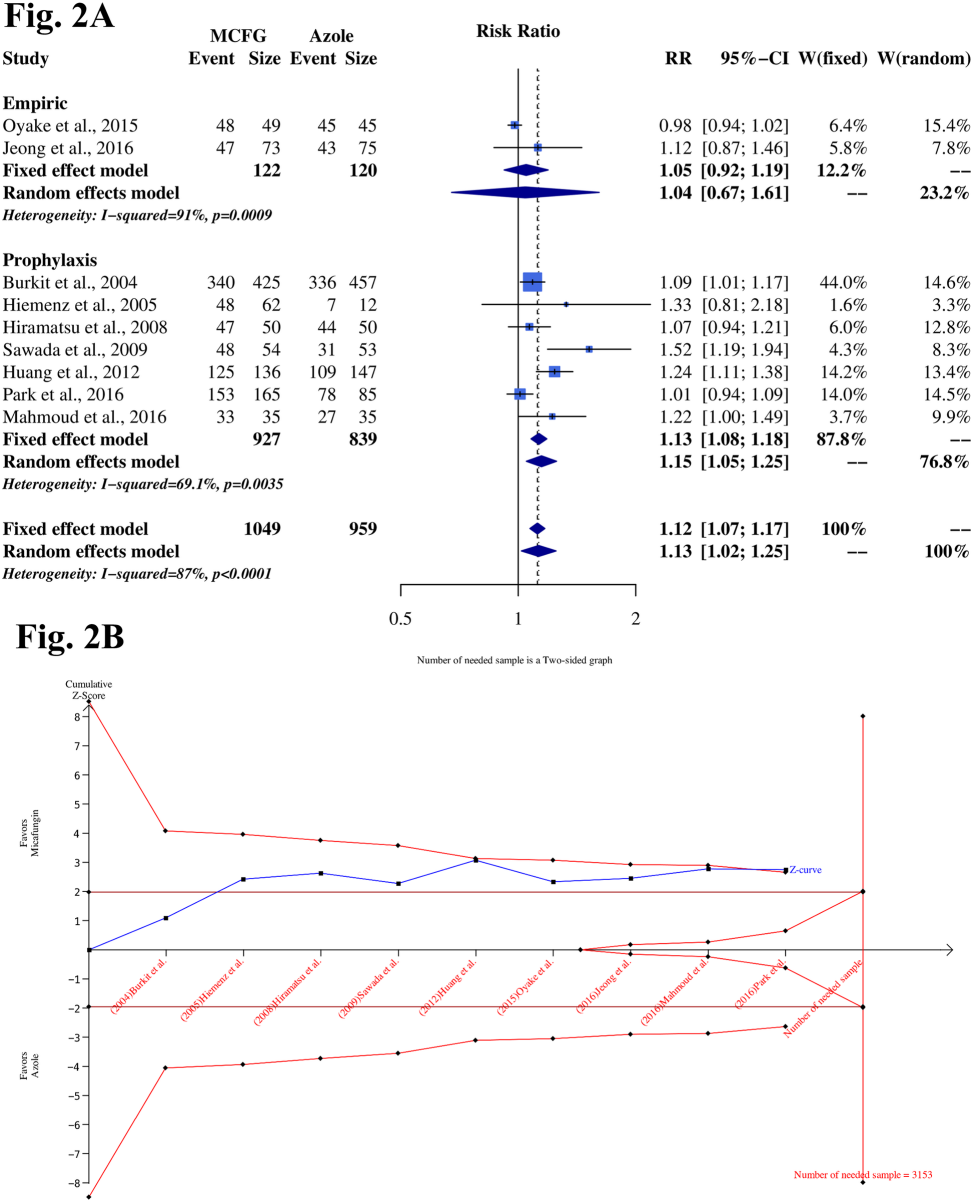
The treatment success rate result of MCFG compared with triazoles. **Fig 2A Treatment success rate from subgroup analysis of prophylaxis and empiric treatment. Prophylaxis**: Initiated at a period of high risk of infection to prevent fungal infections. **Empirical treatment**: Initiation or modification of an existing anti-fungal regimen in persistently febrile patients with neutropenia (generally 4–7 days in duration) that is without a known source and is unresponsive to appropriate antibacterial agents. **Fig 2B Trial sequential analysis (TSA) of 9 trials comparing treatment success rates of MCFG and triazoles**. Heterogeneity adjusted required information size of 3153 participants calculated on basis of proportion of treatment success rates of 80% in triazoles group, relative risk reduction of 20%, α = 5%, β = 20%, Power = 0.95, and I2 = 87%. Cumulative Z-curve crosses trial sequential monitoring boundary, showing sufficient evidence reached for 20% increase in relative risk with administered MCFG. Horizontal dark red lines illustrate the traditional level of statistical significance (P = 0.05)

The treatment success rate of all included studies was evaluated in the intention-to-treatment population (N = 2008). Most studies determined efficacy based on the absence of IFIs during the treatment and post-treatment periods. A detailed definition of efficacy is described in Table 1. Due to high statistical heterogeneity, a random-effect model was adopted. The pooled data demonstrated that MCFG had a better treatment success rate than triazoles (RR = 1.13; 95% CI, 1.02–1.25; I^2^ = 87.0%). Fig 2A provides details on the subgroup analysis of empiric and prophylaxis models, which showed that MCFG was associated with a significantly higher treatment success rate than triazoles for the prophylaxis model in patients undergoing intensive chemotherapy or SCT (RR = 1.15; 95% CI, 1.05––1.25, I^2^ = 69.1%). The empiric model shows no difference between groups (RR = 1.04; 95% CI, 0.67–1.61, I^2^ = 91%). However, publication bias was suspected based on the Egger’s test (p < 0.05). Later, we conducted further assessments and reported no publication bias with actual heterogeneity [33].

In order to analyze the treatment success rate, the trial sequencing monitoring boundaries applied to the meta-analysis assumed a decrease in relative risk of 20%. The cumulative Z-curve crossed the trial sequential monitoring boundary for benefit, indicating that sufficient evidence exists for a 20% relative risk reduction (RRR) when MCFG is administrated (Fig 2B).

#### 3.3.2 Invasive fungal infections

**Fig 3:**
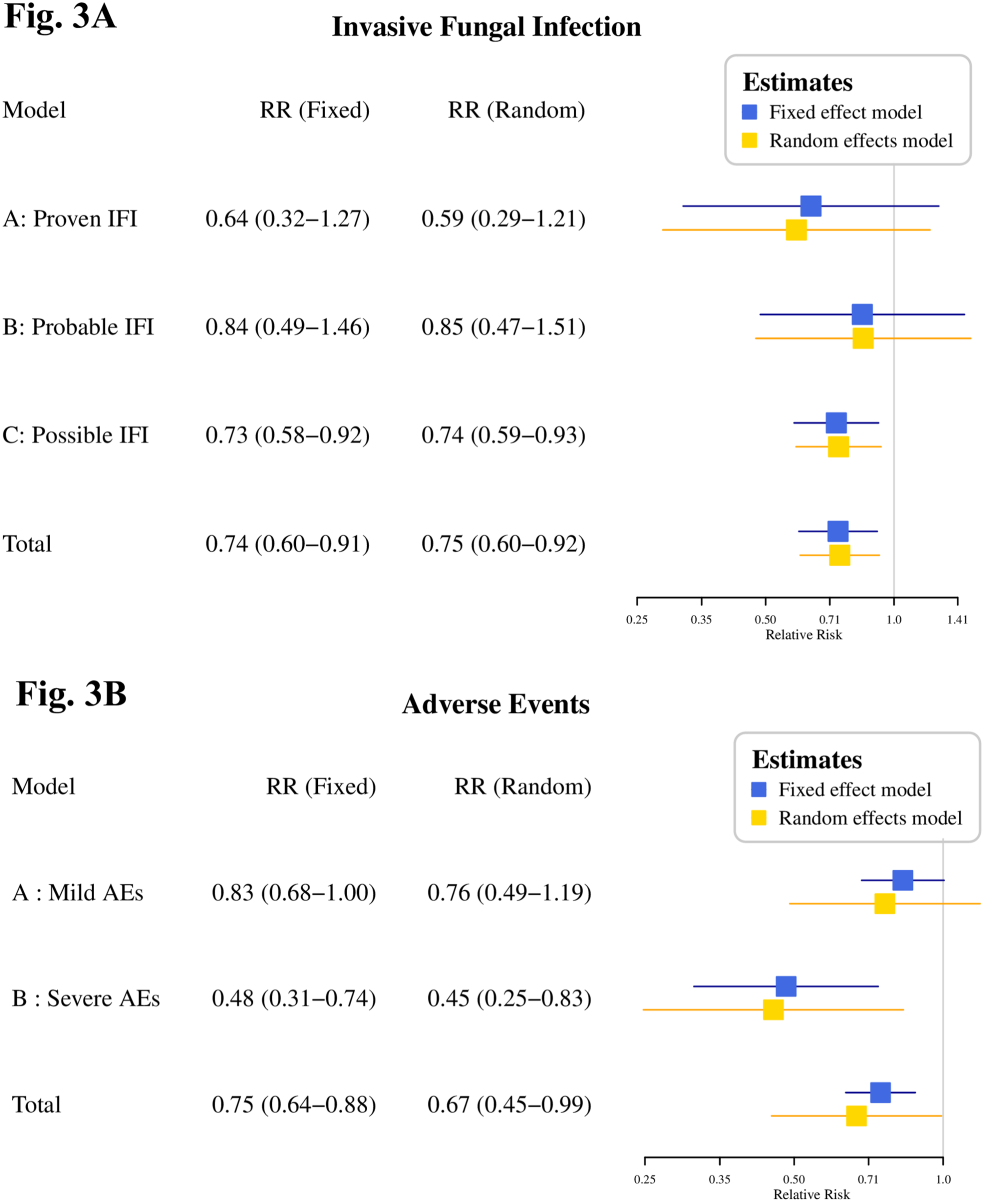
Forest plots of main outcomes compared MCFG with triazoles. **Fig 3A Selected results from the meta-analysis of proven, probable and possible Invasive Fungal Infections with MCFG compared to triazoles**. Forest plot of the relative risks of IFIs, which were divided into proven, probable and possible IFIs in the MCFG and triazoles groups. We found a significantly lower incidence of overall IFIs and possible IFIs, but no difference in proven IFIs and probable IFIs. **Fig 3B Selected side effect results from the meta-analysis of mild and severe adverse events of MCFG compared to triazoles**. Forest plot of the relative risks of AEs, which were divided into mild (grade 1–2) and severe (grade 3–4) AEs in the MCFG and triazoles groups. We found significantly lower incidence of overall AEs and severe AEs in the MCFG group, but no difference when comparing mild AEs.

Analysis of the pooled data reported that MCFG had a significantly lower incidence of overall IFIs (RR = 0.75; 95% CI, 0.61–0.92; I^2^ = 2.1%). This analysis comprised of 3 models, which were classified as proven, probable, or possible. The RR was 0.59 (95% CI 0.29–1.21; I^2^ = 0%), 0.85 (95% CI 0.47–1.51; I^2^ = 0%) and 0.74 (95% CI 0.59–0.93; I^2^ = 0%), respectively (Fig 3A). The pooled RRs demonstrated that MCFG was not superior to any triazoles in cases of breakthrough infection (for both the proven and probable models). MCFG only demonstrated a significantly lower incidence of IFIs (P < 0.05) in the possible model (Table 2). There was no significant publication bias detected among the studies according to the Egger’s test (p>0.05) (S4B Fig)

**Table 2:** Effects (Relative risk) of micafungin on clinical outcomes in patients with febrile neutropenia under intensive chemotherapy or stem cell transplant.

Outcome assessment	No. of trials (patients)	Risk Ratio (95% CI) Fixed-Effect estimate	Risk Ratio (95% CI) Random-Effect estimate	P value Random-Effect estimate	Heterogeneity I^2^(%), Cochrane Q (p-value)	P value of Egger's test
**Treatment Success Rate**	9 (2008)	1.120 (1.072–1.170)	1.129 (1.019–1.250)	**0.0205** ^a^	87.00%,P<0.0001	**0.0141** ^a^
**Fungal infection, Overall**	9 (2008)	0.739 (0.607–0.900)	0.748 (0.609–0.919)	**0.0056** ^a^	2.10%,P = 0.4166	0.4893
**IFI, Proven**	9 (2008)	0.639 (0.320–1.273)	0.590 (0.287–1.214)	0.1521	0.00%,P = 0.6426	0.8969
**IFI, Probably**	9 (2008)	0.843 (0.486–1.462)	0.847 (0.474–1.512)	0.5742	0.00%,P = 0.7025	0.3232
**IFI, Possible**	9 (2008)	0.732 (0.583–0.920)	0.741 (0.589–0.932)	**0.0105** ^a^	0.00%,P = 0.5483	0.6449
**Change of systematic anti-fungal agents**	8 (1901)	0.827 (0.750–0.913)	0.662 (0.466–0.940)	**0.0210** ^a^	71.50%,P = 0.0009	0.0678
**Discontinued prematurely**	7 (1831)	0.523 (0.402–0.680)	0.512 (0.344–0.762)	**0.0010** ^a^	46.80%,P = 0.0940	0.4446
**All-cause mortality**	8 (1901)	0.741 (0.507–1.083)	0.759 (0.515–1.118)	0.1624	0.00%,P = 0.7875	0.9771
**Adverse Event, Overall**	9 (1938)	0.748 (0.632–0.886)	0.655 (0.416–1.032)	0.0682	81.50%,P<0.0001	0.1849
**AEs, Mild**	9 (1938)	0.821 (0.678–0.994)	0.746 (0.472–1.177)	0.2078	74.60%,P = 0.0005	0.2958
**AEs, Severe**	9 (1938)	0.482 (0.314–0.739)	0.454 (0.248–0.831)	**0.0105** ^a^	34.10%,P = 0.1805	0.8899
**AEs, Allergic reaction**	6 (1581)	0.852 (0.485–1.497)	0.672 (0.216–2.092)	0.4923	67.00%,P = 0.0281	0.0658
**AEs, Hepatic function**	5 (1507)	0.713 (0.517–0.983)	0.700 (0.502–0.978)	**0.0363** ^a^	3.30%,P = 0.3881	0.5539
**AEs, Neurologic symptoms**	2 (168)	0.172 (0.049–0.595)	0.183 (0.058–0.572)	**0.0035** ^a^	0.00%,P = 0.7943	NA
**AEs, Electrolyte imbalance**	4 (1413)	1.362 (0.870–2.134)	1.280 (0.812–2.017)	0.2875	0.00%,P = 0.4467	0.1648
**AEs, GI upsets**	2 (1165)	0.623 (0.423–0.917)	0.622 (0.422–0.917)	**0.0164** ^a^	0.00%,P = 0.8438	NA

CI: confidence interval; IFI: Invasive fungal infection; AE: Adverse event; GI: Gastro-intestinal

I^2^: index for assessing heterogeneity; value >50% indicates a moderate to high heterogeneity.

Egger's test: p value of Egger's regression for asymmetry assessment.

^a^: The significance level in the classical model was set as <0.05

#### 3.3.3 Rotation of anti-fungal agents

The concerns associated with the rotation of anti-fungal agents are mainly attributed to potential IFIs or lack of efficacy. From an analysis of 8 pooled trials (N = 1901), MCFG demonstrated a significantly lower incidence of infection than triazoles (RR = 0.66; 95% CI, 0.47–0.94; 71.5%).(S2A Fig)

#### 3.3.4 Overall mortality

No significant difference was found in the all-cause mortality rate. Among the 8 studies, the relative risk of mortality was 0.76 (95% CI 0.52–1.12; I^2^ = 0%). (S2C Fig). And there was no significant publication bias (S4C Fig).

### 3.4 Safety

#### 3.4.1 Premature discontinuation

Premature discontinuation of anti-fungal agents was attributed to lack efficacy or toxicity, based on the physician’s judgment or patient’s concern. 7 studies reported that the pooled RR was 0.51 (95% CI 0.34–0.76; I^2^ = 46.8%), demonstrating that MCFG had a significantly lower incidence of premature discontinuation events (P < 0.05). (S2B Fig)

#### 3.4.2 Adverse events

All trials recorded AEs, however, the majority of trials did not focus on drug-related AEs. The extracted data showed that MCFG did not demonstrate any difference in overall AEs (RR = 0.65; 95% CI 0.41–1.03; I^2^ = 81.5%) when compared to the triazoles group. Furthermore, there was no significant publication bias (S4D Fig). When AEs were classified as mild (grade 1–2) or severe (grade 3–4), the estimated incidence was 0.74 (95% CI 0.47–1.17; I^2^ = 74.6%) and 0.45 (95% CI 0.25–0.83; I^2^ = 34.1%), respectively (Fig 3B).

Meanwhile, a subgroup analysis of AEs revealed that pooled estimates for the incidence of allergic reaction, hepatic impairment, neurologic complications, electrolyte imbalance and gastro-intestinal upset were 0.67 (95% CI 0.22–2.09; I^2^ = 67.0%), 0.70 (95% CI 0.50–0.98; I^2^ = 3.3%), 0.18 (95% CI 0.06–0.57; I^2^ = 0%), 1.28 (95% CI 0.81–2.02; I^2^ = 0%) and 0.62 (95% CI 0.42–0.92; I^2^ = 0%), respectively (further details are provided in Table 2).

MCFG clearly demonstrated a lower incidence of severe AEs (specifically in relation to hepatic impairment, neurologic complications and gastro-intestinal upset) when compared with triazoles, and premature discontinuation events were comparable (Table 2).

### 3.5 Publication bias and sensitivity analysis

#### 3.5.1 Publication bias

**Fig 4:**
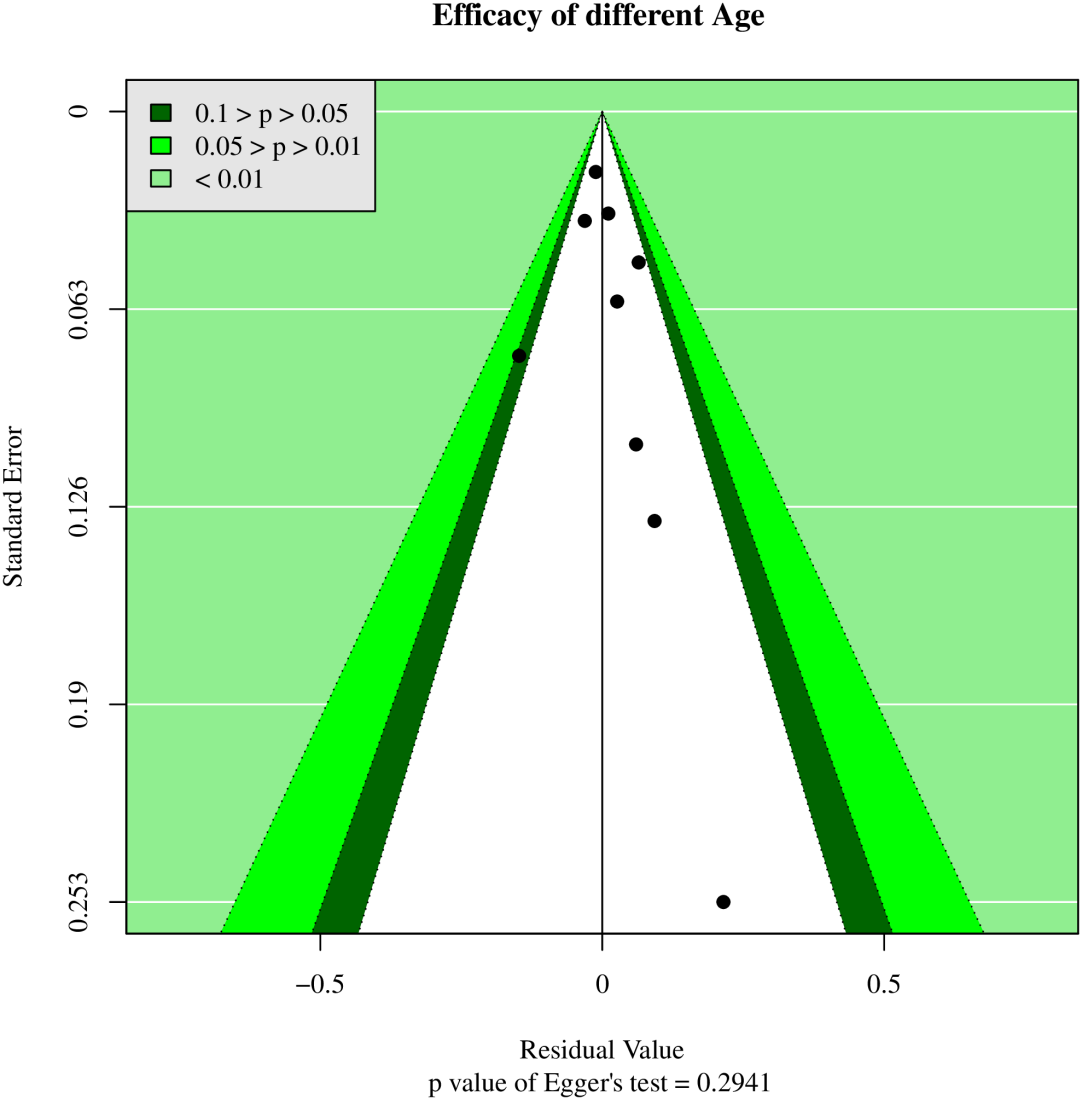
Funnel plot of actual heterogeneity in treatment efficacy model. Analysis of treatment success rates used the Egger’s test to assess actual heterogeneity. No statistical heterogeneity was found (p-value of Egger's test = 0.2941).

To assess potential publication bias, we analyzed funnel plots and Egger’s regression models. Funnel plots were used to demonstrate the association between RRs and standard error for anti-fungal agents, with each plot point representing a study. In regards to the treatment success outcome, we initially found that Egger’s regression yielded potential publication bias (Table 2, S4A Fig). However, further analysis of treatment success rates yielded no statistical heterogeneity (p-value of Egger's test = 0.2941) (Fig 4) [33]

#### 3.5.2 Sensitivity analysis and meta-regression

**Fig 5:**
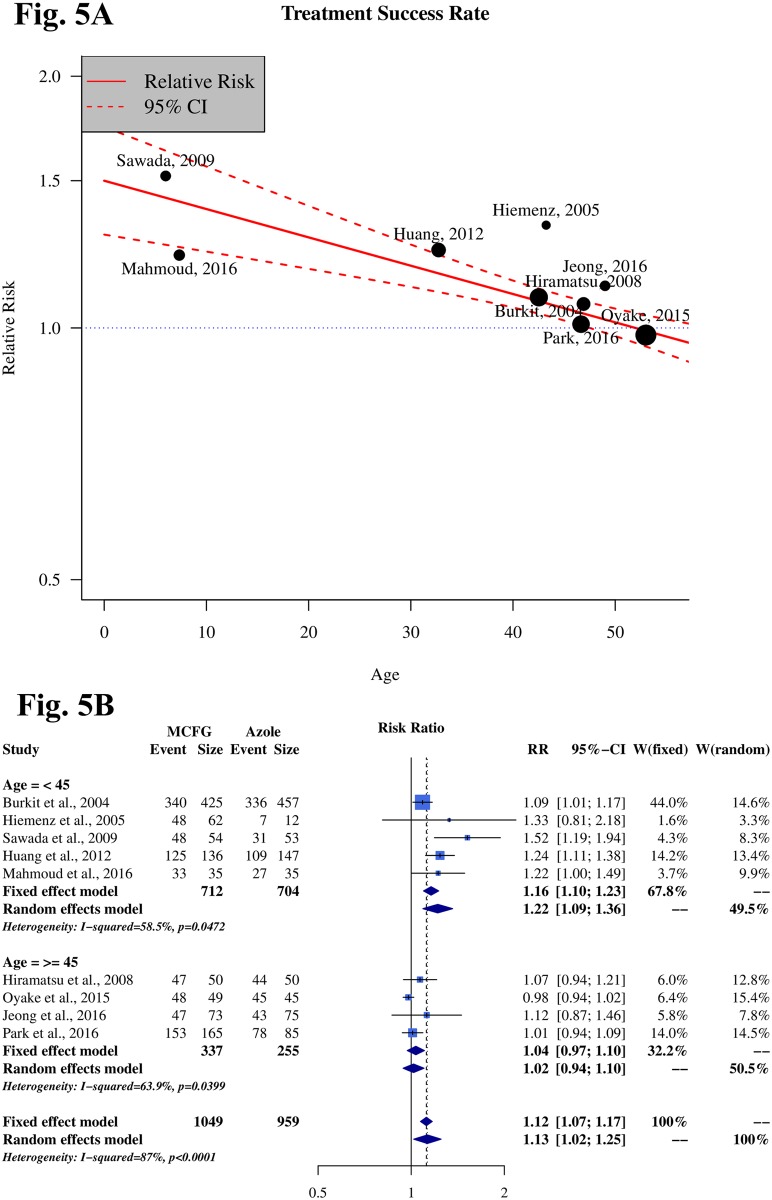
Meta-regression plot and forest plot of treatment success rates and mean age. **Fig 5A Meta-regression plot correction of mean age and treatment success rate**. From the meta-regression plot correction, we determined that younger age correlated with higher efficacy. The point of determination for difference in efficacy is about 45 years old. **Fig 5B Treatment success rate from subgroup analysis of mean age, with patients younger and older than 45 years**.

Table 3 shows the results of a meta-regression that explored the source of high heterogeneity (p < 0.1). All potential factors (S4 Table) could not significantly explain heterogeneity in the meta-analyses of all anti-fungal agents and in the post-hoc analysis, with the exception of mean age. Meta-regression analysis demonstrated a statistically significant correlation between mean age and treatment success rate (P<0.0001). We plotted this finding to disclose the correction (Fig 5A). From the meta-regression result, we conducted a subgroup analysis with groups of patients younger or older than 45 years. This subgroup analysis demonstrated a significantly lower heterogeneity value in each group (RR = 1.22; 95% CI 1.09–1.36; I^2^ = 58.5%) and (RR = 1.02; 95% CI 0.94–1.10; I^2^ = 63.9%, respectively) (Fig 5B), which suggests that MCFG has stronger efficacy in the population younger than 45 years old.

**Table 3:** Meta-regression analysis of heterogeneity.

Moderators	Variables	Study Number (N)	RR_interaction_ (95% CI)	P-value	Cochrane Q p-value	I^2^ (%)
**Treatment Success Rate**	Underlying Therapy (SCT or C/T)	9	0.695 (0.511 to 0.947)	0.0209	0.0200	60.08%
	Quality (Risk of Bias score)	9	0.991 (0.939 to 1.047)	0.7552	0.0002	74.78%
	Mean Age (all population)	9	0.992 (0.989 to 0.995)	***<0*.*0001***^a^	0.5405	0.00%
	Allogenic transplant (%)	6	0.999 (0.998 to 1.001)	0.4671	0.0021	76.23%
	Neutropenia duration	5	0.980 (0.942 to 1.020)	0.3296	0.0426	63.30%
	Underlying Leukemia (%)	8	0.960 (0.698 to 1.321)	0.8034	0.0004	75.49%
**Discontinued Prematurely**	Underlying Therapy (SCT or C/T)	7	1.406 (0.659 to 2.999)	0.3780	0.1779	34.46%
	Quality (Risk of Bias score)	7	1.186 (0.896 to 1.571)	0.2335	0.2198	28.68%
	Mean Age (all population)	7	1.025 (0.970 to 1.083)	0.3794	0.1672	36.59%
	Allogenic transplant (%)	5	1.003 (0.998 to 1.009)	0.2326	0.8323	0.00%
	Neutropenia duration	5	0.939 (0.723 to 1.218)	0.6353	0.0412	63.62%
	Underlying Leukemia (%)	6	1.745 (0.260 to 11.703)	0.5666	0.0935	49.67%
**AEs, Overall**	Underlying Therapy (SCT or C/T)	7	0.973 (0.321 to 2.948)	0.9616	<0.0001	80.95%
	Quality (Risk of Bias score)	7	1.036 (0.752 to 1.427)	0.8297	0.0001	78.44%
	Mean Age (all population)	7	1.030 (0.957 to 1.110)	0.4261	<0.001	78.92%
	Allogenic transplant (%)	5	1.000 (0.994 to 1.007)	0.8964	0.0409	59.88%
	Neutropenia duration	5	0.835 (0.609 to 1.145)	0.2621	<0.001	88.52%
	Underlying Leukemia (%)	7	0.657 (0.088 to 4.921)	0.6824	<0.001	81.04%
**AEs, Mild**	Underlying Therapy (SCT or C/T)	8	0.592 (0.103 to 3.399)	0.5565	0.0027	72.60%
	Quality (Risk of Bias score)	8	1.098 (0.775 to 1.554)	0.5987	0.0012	72.67%
	Mean Age (all population)	8	0.992 (0.959 to 1.026)	0.6397	0.0004	75.75%
	Allogenic transplant (%)	6	0.999 (0.993 to 1.006)	0.8600	0.0268	63.55%
	Neutropenia duration	5	0.827 (0.549 to 1.244)	0.3614	<0.001	87.23%
	Underlying Leukemia (%)	7	0.825 (0.083 to 8.148)	0.8689	0.0004	77.69%
**AEs, Severe**	Underlying Therapy (SCT or C/T)	8	2.529 (0.420 to 15.247)	0.3113	0.2897	19.01%
	Quality (Risk of Bias score)	8	0.853 (0.522 to 1.395)	0.5276	0.2823	19.33%
	Mean Age (all population)	8	1.020 (0.983 to 1.058)	0.2867	0.3685	7.83%
	Allogenic transplant (%)	6	1.001 (0.990 to 1.011)	0.9123	0.1945	34.02%
	Neutropenia duration	5	0.866 (0.639 to 1.174)	0.3537	0.2760	22.45%
	Underlying Leukemia (%)	7	0.917 (0.095 to 8.870)	0.9407	0.2413	25.74%
**AEs, Allergic Reaction**	Underlying Therapy (SCT or C/T)	6	1.046 (0.108 to 10.144)	0.9687	0.0590	55.98%
	Quality (Risk of Bias score)	6	1.429 (0.670 to 3.047)	0.3556	0.1372	42.66%
	Mean Age (all population)	6	0.950 (0.779 to 1.159)	0.6118	0.0783	52.32%
	Allogenic transplant (%)	4	1.007 (0.999 to 1.016)	0.0946	0.7429	0.00%
	Neutropenia duration	5	0.736 (0.533 to 1.016)	0.0624	0.4383	0.00%
	Underlying Leukemia (%)	5	0.465 (0.005 to 44.446)	0.7420	0.1095	50.37%

Treatment: intensive chemotherapy (C/T) or stem cell transplant (SCT); C/T is reference.

RR_interaction_: interaction effect calculated by meta-regression; positive direction indicates that possible moderators might strengthen the treatment success rate in Micafungin relative to extensive Azole medication.

^a^:The significant level was set as 0.05.

In the meta-regression analysis, mean age and triazoles model were the only 2 significant moderators for exploring the heterogeneity of efficacy (p-value = 0.0007651435). We attempted to further separate their effects, but they were both insignificant in multivariable meta-regression (p-value = 0.735474; 0.2017).

### 3.6 Stratified analysis

Stratified analysis was conducted by creating 1 group for each of the 3 triazoles, FLCZ, ITCZ and VOCZ. In regards to treatment success rate, MCFG had better efficacy, and the RRs were 1.11 (95% CI 1.00–1.23; I^2^ = 67.8%), 1.22 (95% CI 1.11–1.35; I^2^ = 0%) and 1.09 (95% CI 0.70–1.70; I^2^ = 94.9%), respectively (S3A Fig). Overall, IFIs incidence was 0.71(95% CI 0.56–0.90; I^2^ = 0%), 0.98 (95% CI 0.66–1.44; I^2^ = 0%) and 0.28 (95% CI 0.09–0.80; I^2^ = 0%), respectively (S3 Fig). Overall, AEs incidence was 0.80 (95% CI 0.64–1.01; I^2^ = 0%), 0.62 (95% CI 0.14–2.81; I^2^ = 95.1%) and 0.33 (95% CI 0.18–0.60; I^2^ = NA), respectively (S3C Fig). The details of other stratified analyses are recorded in Table 4.

**Table 4:** Effects of stratified analyses.

Moderators	No. of trials (patients)	RR_interaction_ (95% CI)	Heterogeneity I^2^(%)	P value
**Treatment success rate**				0.4063
Fluconazole	5 (1413)	1.110 (1.002–1.230)	67.80%	
Itraconazole	2 (431)	1.221 (1.106–1.349)	0.00%	
Voriconazole	2 (164)	1.088 (0.695–1.704)	94.90%	
**Fungal infection, overall**				0.068
Fluconazole	5 (1413)	0.71 (0.56–0.90)	0.00%	
Itraconazole	2 (431)	0.98 (0.66–1.44)	0.00%	
Voriconazole	2 (164)	0.28 (0.09–0.80)	0.00%	
**Change of systematic Anti-fungal agents**				0.1469
Fluconazole	5 (1306)	0.867 (0.709–1.060)	7.20%	
Itraconazole	2 (431)	0.984 (0.725–1.337)	0.00%	
Voriconazole	2 (164)	0.213 (0.046–0.984)	77.40%	
**AEs, Overall**				0.0213
Fluconazole	5 (1413)	0.805 (0.640–1.011)	0.00%	
Itraconazole	2 (431)	0.618 (0.136–2.807)	95.10%	
Voriconazole	1 (94)	0.328 (0.180–0.596)	NA	
**AEs, Mild**				0.0528
Fluconazole	5 (1413)	0.910 (0.705–1.174)	0.00%	
Itraconazole	2 (431)	0.694 (0.133–3.621)	95.20%	
Voriconazole	1 (94)	0.328 (0.129–0.838)	NA	
**AEs, Severe**				0.4733
Fluconazole	4 (1413)	0.44 (0.141–1.345)	49.60%	
Itraconazole	1 (176)	0.665 (0.335–1.321)	NA	
Voriconazole	1 (94)	0.328 (0.128–0.838)	NA	

## Discussion

IFIs are now the leading cause of mortality and morbidity in patients undergoing intensive chemotherapy or SCT. Early diagnosis of IFIs is still extremely challenging. As a result, prevention and treatment with anti-fungal agents is crucial for these patients. MCFG’s ability to fight against invasive candidiasis and invasive mold infections has recently been discovered. However, there is still a lack of sufficient evidence to prove MCFG’s efficacy and triazoles’ safety.

Our meta-analysis comprised of all RCTs conducted through November 2016. TSA revealed that our pooled sample size had sufficient power in comparison MCFG with extended-spectrum Azole for the result of treatment success rate, which means based on current evidence we could assume MCFG did superior to other Azoles in efficacy outcome. The main outcome of our analysis demonstrates that MCFG had higher efficacy and less severe AEs than triazoles, and was compatible with previous articles [12, 17]. Meanwhile, we disclosed stronger evidence of the correction between the mean age and treatment success rate, which had been mentioned in the Park, J. S. 2010 study [34]. Nevertheless, there was no convincing hypothesis could explain the association. We considered this point is needed further research for proving this new phenomenon.

For analysis of treatment success rates, treatment success was defined as the absence of IFIs during treatment and 4 weeks post-treatment. 2 studies took safety factors into consideration, including early discontinuation due to severe AEs or lack of efficacy.

The meta-analysis conducted by Wang, J.F., 2016 suggested that there was no significant difference in efficacy for the treatment of candidemia or invasive candidiasis between echinocandins and comparator triazoles. However, there were significantly positive outcomes for the prevention model [35]. Our pooled analysis of 9 RCTs with a total of 2,008 patients confirmed that MCFG had a higher treatment success rate for the prevention model, but not for the empiric treatment model. The prophylaxis efficacy reported in the Kobayashi, C., 2015 post-marketing surveillance study was 72.8%. However, our data demonstrated a higher efficacy (84.7%), which may be attributed to a difference in the percentage of high risk populations such as allogenic HST patients (63.1% vs 43.0%) [36]. Among our study, the subgroup analyses indicated that MCFG possessed a higher treatment success rate for the prophylactic therapy of fungal infections when compared to triazoles. Furthermore, we conducted stratified analyses of different triazole models, MCFG yielded both statistically significant superiority when compared to FLCZ and ITCZ.

Classification criteria for possible, probable, and proven IFIs were based on 2008 criteria provided by the EORTC and MSG [37]. There was no significant difference between the incidences of proven or probable IFIs. However, the incidence of possible IFIs was significantly lower when MCFG was administered compared to different triazoles. Further stratified analyses revealed MCFG prevented IFIs more effectively than FLCZ and VOCZ with low heterogeneity. The *XU*, *S*.*X*., *2016* also reported similar results about the comparison of MCFG to comparative anti-fungal agents (including FLCZ, ITCZ, caspofungin and placebo) [38].

Usually, patients who undergo prophylactic anti-fungal agent rotate among different anti-fungal agents while there is a suspicion of possible IFIs or if the currently administered anti-fungal agent lacks efficacy. We assessed the difference between fixed effect estimates and random effects estimates, and found that the outcomes of rotating systematic anti-fungal agents had a large variability. Furthermore, this type of treatment had high heterogeneity and borderline significant asymmetry. Fortunately, the estimated results obtained by fixed effect assumption and random effect assumption were both significant, so the clinical interpretation were not impacted. The pooling comparison indicated MFCG had significantly lower rotating incidence than other azoles.

AEs are another important issue. It is difficult to determine the association between anti-fungal drugs and AEs. According to the Kobayashi, C., 2015 report, the occurrence rate of overall AEs was up to 59% with 3.7% of AEs being classified as serious. Our pooled results had a much lower incidence of overall AEs (18.2%), but a similar occurrence of severe AEs (2.7%) [36]. From the meta-analysis, we found MCFG had significantly lower incidence of severe adverse events and hepatic impairment events (S2D Fig). MCFG is known to have little host toxicity in clinical practice [39]. Hepatotoxicity is a common drug related AE in anti-fungal agents. Wang 2010 [40] conducted a meta-analysis about Tolerability and hepatotoxicity in different anti-fungal agents which revealed MCFG and FLCZ had similar lower hepatic impairment events (2.0–9.3%) than ITCZ and VOCZ. MCFG had significantly lower hepatotoxicity may be attributed from Jeong 2016 study which takes a large weight proportion in pooling meta-analysis.

In regards to premature discontinuation and changing to other systemic anti-fungal agents, MCFG had significantly lower incidence when compared to all triazoles. This result was compatible with MCFG’s lower incidence of severe AEs. Additional RCTs are needed to assess drug-related AEs.

We conducted a TSA to calculate the sample size required to assess this issue [24]. The monitoring boundaries used were as follows: a two-sided test with a power = 0.95 (we used a higher power to avoid false negatives) at a significance level of 0.05, ratio of controls to cases = 80.1% (based on data), and I^2^ (heterogeneity) = 87%. Following these settings, the number of required samples is equal to 3153 and we collected 2008 samples in this meta-analysis. The cumulative Z-curve crossed the trial sequential monitoring boundary, demonstrating sufficient evidence for a 20% decrease in relative risk with administrated MCFG. This may increase confidence in our result. MCFG has higher treatment success rates.

Some limitations still existed in our meta-analysis. First, some clinical heterogeneity was attributed to the use of different comparative triazoles. Additional heterogeneity may stem from the collective pooling of prophylaxis and treatment trials, even though a meta-regression test reported no statistic heterogeneity. These may in fact lead to some clinically significant confounding effects. Second, the majority of studies compared MCFG to FLCZ (N = 5). Only 2 studies compared MCFG to ITCZ, and only 2 compared MCFG to VOCZ. Although MCFG demonstrated similarly higher treatment success rated when compared to FLCZ and ITCZ, the limited population may have induced selection bias. Meanwhile, we did not find any RCTs that evaluated the association between MCFG and posaconazole. Third, the enrolled patients in the majority of trials were at high risk of mortality (leukemia accounted for at least 50% of the population) due to underlying illness. When we assessed the all-cause mortality, we determined that many patients might have died due to an underlying disease instead of an AE caused by anti-fungal drugs. Fourth, there were only 2 trials with a pediatric population [7, 31], which may have caused some selection bias. Despite these limitations, our results documented the comparable effectiveness of systemic anti-fungal drugs and identified notable differences between MCFG and triazoles, with a summary of all evidence in a single descriptive table (Table 3).

In conclusion, despite the above limitations, our results suggested that MCFG is as effective as triazoles for the prophylaxis and treatment of patients with fungal infections. Furthermore, the treatment success rate of MCFG was superior to that of triazoles for the prophylaxis model. Stronger efficacy was demonstrated when mean age was corrected for, especially for patients younger than 45 years old. Compared to triazoles, MCFG was safer in that it demonstrated a lower rate of severe AEs including hepatotoxicity, which may be responsible for the MCFG group’s lower premature discontinuation rate. This meta-analysis shows that MCFG appears to be well-tolerated with manageable side effects and positive efficacy in the prophylaxis of IFIs. Further large scale, high quality RCTs should be conducted to assess the effects of drug-related AEs and establish the best definite regimen of treatment.

## Supporting information

S1 Table Search strategies and detailed records. (PDF)

S2 Table PRISMA 2009 checklist. (PDF)

S3 Table Detailed quality assessment. (PDF)

S4 Table Detailed characteristics and available data of included studies. (PDF)

S1 Fig Summary of included trials risk of bias. (A) Risk of bias graph. (B) Risk of bias summary.
(TIFF)

S2 Fig Forest plots of the selected results. (A) Forest plot of rotation of anti-fungal agents model. (B) Forest plot of prematurely discontinued model. (C) Forest plot of All-Caused Mortality model. (D) Forest plot of Adverse Events, Hepatic impairment model.
(PDF)

S3 Fig Forest plots of stratified analyses for of selective results. (A) Forest plots of Treatment Success Rates model. (B) Forest plots of Fungal Infection, Overall model. (C) Forest plots of Adverse Events, Overall model.
(PDF)

S4 Fig Funnel plots of the selected results. (A) Funnel plot of Treatment Success Rates model. (B) Funnel plot of Fungal Infection, Overall model. (C) Funnel plot of All-Cause Mortality model. (D) Funnel plot of Adverse Events, Overall model.
(PDF)
