# Supplementary material for: Efficacy and safety of micafungin versus extensive azoles in the prevention and treatment of invasive fungal infections for neutropenia patients with hematological malignancies: A meta-analysis of randomized controlled trials
Source: PLoS One. 2017 Jul 12;12(7):e0180050. doi: 10.1371/journal.pone.0180050 (PMC5507498; doi:10.1371/journal.pone.0180050)
Supplement: S1 Table — (PDF) [file pone.0180050.s001.pdf]

S1 Table. **Search strategies and detailed records.**

Relevant text of Micafungin	Relevant text of febrile neutropenia
1. Micafungin	10. Febrile neutropenia
2. Micafungin sodium	11. Neutropenic fever
3. Micamine	12. Febrile neutropenias
4. FK 463	13. Neutropenia, fever
5. FK-463	14. Chemo-induced febrile neutropenia
6. Echinocandin	15. 10 or 11 or 12 or 13 or 14
7. Lipopeptides	**Combined (Final strategy)**
8. Antifungal prophylaxis	16. 9 and 15
9. (1 or 2 or 3 or 4 or 5) or ((6 or 7) and (9))	

Web sites and uniform resource locator:

PUBMED: http://www.ncbi.nlm.nih.gov/pubmed

EMBASE: https://www.embase.com

COCHRANE CENTRAL: https://www.cochrane.com

Records from EMBASE [1-71] :
Unrelated records (wrong outcome [3-5,7,8,10-13,15-17])
Included studies [41,42,45,47,70]

Records from PUBMED [2,3,30,36,42,45,47,66,72-80, 86,92] (8 of record is a duplicate record [1]):
Unrelated records (wrong outcome [73-76,78-80])
Included studies [77,92]

Records from Cochrane central[1,2,6,16,19,20,21,28,44,62,63,81-100] (11 of record is a duplicate record [1], 4 of record is a duplicate record [2]):
Unrelated records (wrong outcome [81-83, 85-91,93-100])
Included studies [84]

Additional Records from Google scholar [101]
Included studies [101]

Reference

1. Micafungin: new drug. Severe candidiasis: a third echinocandin, with life-threatening hepatotoxicity. Prescrire international. 2009;18(102):154.

2. Multicenter comparison of the etest and EUCAST methods for antifungal susceptibility testing of Candida isolates to micafungin. Antimicrobial Agents and Chemotherapy 60 (8) (pp 5088-5091), 2016 Date of Publication: August 2016 [Internet]. 2016. Available from: http://onlinelibrary.wiley.com/o/cochrane/clcentral/articles/655/CN-01197655/frame.html.

3. Agarwal R, Vishwanath G, Aggarwal AN, Garg M, Gupta D, Chakrabarti A. Itraconazole in chronic cavitary pulmonary aspergillosis: A randomised controlled trial and systematic review of literature. Mycoses. 2013;56(5):559-70.

4. Ahn JS, Kim YK, Min YH, Cheong JW, Jang JH, Jung CW, et al. Azacitidine Pre-Treatment Followed by Reduced-Intensity Stem Cell Transplantation in Patients with Higher-Risk Myelodysplastic Syndrome. Acta haematologica [Internet]. 2015; 134(1):[40-8 pp.]. Available from: http://onlinelibrary.wiley.com/o/cochrane/clcentral/articles/574/CN-01085574/frame.html.

5. Alexander BD. Prophylaxis of invasive mycoses in solid organ transplantation. Current Opinion in Infectious Diseases. 2002;15(6):583-9.

6. Alvarez-Lerma F, Grau S, Lopez C, Jimenez JD, Trasmonte MV, Nieto M, et al. Patients treated with micafungin during their stay in intensive care unit. [Spanish]. Medicina Intensiva [Internet]. 2015; 39(8):[467-76 pp.]. Available from: http://onlinelibrary.wiley.com/o/cochrane/clcentral/articles/230/CN-01128230/frame.html.

7. Andes DR, Reynolds DK, Wart SA, Lepak AJ, Kovanda LL, Bhavnani SM. Clinical pharmacodynamic index identification for micafungin in esophageal candidiasis: dosing strategy optimization. Antimicrobial agents and chemotherapy [Internet]. 2013; 57(11):[5714-6 pp.]. Available from: http://onlinelibrary.wiley.com/o/cochrane/clcentral/articles/938/CN-01121938/frame.html.

8. Bayés M, Rabasseda X, Prous JR. Gateways to clinical trials: November 2006. Methods and Findings in Experimental and Clinical Pharmacology. 2006;28(9):657-78.

9. Bayés M, Rabasseda X, Prous JR. Gateways to clinical trials. Methods and Findings in Experimental and Clinical Pharmacology. 2007;29(7):467-509.
10. Ben-Ami R, Lewis RE, Kontoyiannis DP. Invasive mould infections in the setting of hematopoietic cell transplantation: Current trends and new challenges. Current Opinion in Infectious Diseases. 2009;22(4):376-84.
11. Blyth CC, Palasanthiran P, O'Brien TA. Antifungal therapy in children with invasive fungal infections: A systematic review. Pediatrics. 2007;119(4):772-84.
12. Bossche HV. Echinocandins - An update. Expert Opinion on Therapeutic Patents. 2002;12(2):151-67.
13. Cleary JD. Echinocandins: Pharmacokinetic and therapeutic issues. Current Medical Research and Opinion. 2009;25(7):1741-50.
14. Cornely OA, Herbrecht R, Viscoli C, Greene R, Ruhnke M, Vitek A, et al. Efficacy and safety of micafungin salvage monotherapy vs. active control intravenous monotherapy in patients with invasive aspergillosis. Mycoses [Internet]. 2011; 54:[93-4 pp.]. Available from: http://onlinelibrary.wiley.com/o/cochrane/clcentral/articles/618/CN-01020618/frame.html.
15. Cornely OA, Marty FM, Stucker F, Pappas PG, Ullmann AJ. Efficacy and safety of micafungin for treatment of serious Candida infections in patients with or without malignant disease. Mycoses [Internet]. 2011; 54(6):[e838-47 pp.]. Available from: http://onlinelibrary.wiley.com/o/cochrane/clcentral/articles/326/CN-00811326/frame.html.
16. Cornely OA, Meems L, Herbrecht R, Viscoli C, van Amsterdam RGM, Ruhnke M. Randomised, multicentre trial of micafungin vs. an institutional standard regimen for salvage treatment of invasive aspergillosis. Mycoses. 2015;58(1):58-64.
17. Cornely OA, Sidhu M, Odeyemi I, Van Engen AK, Van Der Waal JM, Schoeman O. Economic analysis of micafungin versus liposomal amphotericin B for treatment of candidaemia and invasive candidiasis in Germany. Current Medical Research and Opinion. 2008;24(6):1743-53.
18. Cornely OA, Vazquez J, Waele J, Betts R, Rotstein C, Nucci M, et al. Efficacy of micafungin in invasive candidiasis caused by common Candida species with special emphasis on non-albicans Candida species. Mycoses [Internet]. 2014; 57(2):[79-89 pp.]. Available from: http://onlinelibrary.wiley.com/o/cochrane/clcentral/articles/186/CN-01001186/frame.html.
19. de Wet N, Llanos-Cuentas A, Suleiman J, Baraldi E, Krantz EF, Della Negra M, et al. A randomized, double-blind, parallel-group, dose-response study of micafungin compared with fluconazole for the treatment of esophageal candidiasis in

HIV-positive patients. Clinical infectious diseases : an official publication of the Infectious Diseases Society of America. 2004;39(6):842-9. Epub 2004/10/09. doi: 10.1086/423377. PubMed PMID: 15472817.
20. De Wet NTE, Bester AJ, Viljoen JJ, Filho F, Suleiman JM, Ticona E, et al. A randomized, double blind, comparative trial of micafungin (FK463) vs. fluconazole for the treatment of oesophageal candidiasis. Alimentary Pharmacology and Therapeutics. 2005;21(7):899-907.
21. Dougherty KA, Bertolaso C, Schall JI, Smith-Whitley K, Stallings VA. Safety and Efficacy of High-dose Daily Vitamin D<inf>3</inf> Supplementation in Children and Young Adults with Sickle Cell Disease. Journal of pediatric hematology/oncology [Internet]. 2015; 37(5):[e308-e15 pp.]. Available from: http://onlinelibrary.wiley.com/o/cochrane/clcentral/articles/387/CN-01164387/frame.html.
22. Dupont B. Micafungin. Journal de Mycologie Medicale. 2010;20(3):194-205.
23. Dupont BF, Lortholary O, Ostrosky-Zeichner L, Stucker F, Yeldandi V. Treatment of candidemia and invasive candidiasis in the intensive care unit: post hoc analysis of a randomized, controlled trial comparing micafungin and liposomal amphotericin B. Critical care (London, England) [Internet]. 2009; 13(5):[R159 p.]. Available from: http://onlinelibrary.wiley.com/o/cochrane/clcentral/articles/651/CN-00730651/frame.html.
24. Estes KE, Penzak SR, Calis KA, Walsh TJ. Pharmacology and antifungal properties of anidulafungin, a new echinocandin. Pharmacotherapy. 2009;29(1):17-30.
25. Falagas ME, Vouloumanou EK, Sgouros K, Athanasiou S, Peppas G, Siempos II. Patients included in randomised controlled trials do not represent those seen in clinical practice: Focus on antimicrobial agents. International Journal of Antimicrobial Agents. 2010;36(1):1-13.
26. Felder S, Grabe K, Mayrhofer T. Micafungin versus caspofungin for the treatment of systemic candida infections: A cost-effectiveness analysis for Germany. [German]. PharmacoEconomics - German Research Articles [Internet]. 2010; 8(2):[71-83 pp.]. Available from: http://onlinelibrary.wiley.com/o/cochrane/clcentral/articles/003/CN-00889003/frame.html.
27. Felder S, Grabe K, Mayrhofer T, Decker S. Micafungin vs caspofungin for the treatment of systemic candida infections: A cost-effectiveness analysis for Germany. Value in Health. 2009;12(7):A426-A7.
28. Felder S, Mayrhofer T. Micafungin versus caspofungin for the treatment of systemic candida infections: A cost-effectiveness analysis for Switzerland. Value in

health [Internet]. 2014; 17(3):[A275 p.]. Available from: http://onlinelibrary.wiley.com/o/cochrane/clcentral/articles/011/CN-01061011/frame.html.
29. Feldmann C. Micafungin is equally effective as liposomal amphotericin in the treatment of invasive candidiasis. Deutsche Medizinische Wochenschrift. 2007;132(21):1144.
30. Fujisawa Y, Oyake T, Sugawara N, Sasaki R, Tsukushi Y, Hanamura I, et al. Comparison of micafungin and liposomal amphotericin b for empirical antifungal therapy in febrile neutropenic patients with hematological malignancies: A randomized controlled trial. Haematologica [Internet]. 2015; 100:[135 p.]. Available from: http://onlinelibrary.wiley.com/o/cochrane/clcentral/articles/012/CN-01092012/frame.html.
31. Gafter-Gvili A, Vidal L, Goldberg E, Leibovici L, Paul M. Treatment of invasive candidal infections: Systematic review and meta-analysis. Mayo Clinic Proceedings. 2008;83(9):1011-21.
32. George J, Reboli AC. Anidulafungin: When and how? The clinician's view. Mycoses [Internet]. 2012; 55(1):[36-44 pp.]. Available from: http://onlinelibrary.wiley.com/o/cochrane/clcentral/articles/500/CN-00889500/frame.html.
33. Glöckner A, Steinbach A, Vehreschild JJ, Cornely OA. Treatment of invasive candidiasis with echinocandins. Mycoses. 2009;52(6):476-86.
34. Golan Y, Harrison D, Fahrbach K. An echinocandin vs. a comparator antifungal in Candida bloodstream infections: A meta-analysis. Clinical Microbiology and Infection. 2010;16:S93.
35. Groll AH, Walsh TJ. Caspofungin: Pharmacology, safety and therapeutic potential in superficial and invasive fungal infections. Expert Opinion on Investigational Drugs. 2001;10(8):1545-58.
36. Hall RG, Swancutt MA, Gumbo T. Fractal geometry and the pharmacometrics of micafungin in overweight, obese, and extremely obese people. Antimicrobial agents and chemotherapy [Internet]. 2011; 55(11):[5107-12 pp.]. Available from: http://onlinelibrary.wiley.com/o/cochrane/clcentral/articles/345/CN-00843345/frame.html.
37. Halton E, Chung D, Xiao K, Quintanilla H, Baldwin C, Baird P, et al. Micafungin versus posaconazole anti-fungal prophylaxis in adult patients with acute leukemia undergoing induction chemotherapy. Blood [Internet]. 2012; 120(21). Available from: http://onlinelibrary.wiley.com/o/cochrane/clcentral/articles/862/CN-01028862/frame.html.

38. Hebert MF, Blough DK, Townsend RW, Allison M, Buell D, Keirns J, et al. Concomitant tacrolimus and micafungin pharmacokinetics in healthy volunteers. Journal of clinical pharmacology [Internet]. 2005; 45(9):[1018-24 pp.]. Available from: http://onlinelibrary.wiley.com/o/cochrane/clcentral/articles/329/CN-00575329/frame.html.
39. Herbrecht R, Flückiger U, Gachot B, Ribaud P, Thiebaut A, Cordonnier C. Treatment of invasive Candida and invasive Aspergillus infections in adult haematological patients. European Journal of Cancer, Supplement. 2007;5(2):49-59.
40. Heresi GP, Gerstmann DR, Reed MD, Anker JN, Blumer JL, Kovanda L, et al. The pharmacokinetics and safety of micafungin, a novel echinocandin, in premature infants. Pediatric infectious disease journal [Internet]. 2006; 25(12):[1110-5 pp.]. Available from: http://onlinelibrary.wiley.com/o/cochrane/clcentral/articles/018/CN-00747018/frame.html.
41. Hiemenz J, Cagnoni P, Simpson D, Devine S, Chao N, Keirns J, et al. Pharmacokinetic and maximum tolerated dose study of micafungin in combination with fluconazole versus fluconazole alone for prophylaxis of fungal infections in adult patients undergoing a bone marrow or peripheral stem cell transplant. Antimicrobial agents and chemotherapy [Internet]. 2005; 49(4):[1331-6 pp.]. Available from: http://onlinelibrary.wiley.com/o/cochrane/clcentral/articles/089/CN-00515089/frame.html.
42. Hiramatsu Y, Maeda Y, Fujii N, Saito T, Nawa Y, Hara M, et al. Use of micafungin versus fluconazole for antifungal prophylaxis in neutropenic patients receiving hematopoietic stem cell transplantation. International journal of hematology. 2008;88(5):588-95.
43. Hope WW, Kaibara A, Roy M, Arrieta A, Azie N, Kovanda LL, et al. Population pharmacokinetics of micafungin and its metabolites M1 and M5 in children and adolescents. Antimicrobial agents and chemotherapy [Internet]. 2015; 59(2):[905-13 pp.]. Available from: http://onlinelibrary.wiley.com/o/cochrane/clcentral/articles/276/CN-01112276/frame.html.
44. Horn DL, Ostrosky-Zeichner L, Morris MI, Ullmann AJ, Wu C, Buell DN, et al. Factors related to survival and treatment success in invasive candidiasis or candidemia: a pooled analysis of two large, prospective, micafungin trials. European journal of clinical microbiology & infectious diseases : official publication of the European Society of Clinical Microbiology [Internet]. 2010; 29(2):[223-9 pp.]. Available from:

http://onlinelibrary.wiley.com/o/cochrane/clcentral/articles/218/CN-00728218/frame.html.
45. Huang X, Chen H, Han M, Zou P, Wu D, Lai Y, et al. Multicenter, randomized, open-label study comparing the efficacy and safety of micafungin versus itraconazole for prophylaxis of invasive fungal infections in patients undergoing hematopoietic stem cell transplant. Biology of blood and marrow transplantation : journal of the American Society for Blood and Marrow Transplantation [Internet]. 2012; 18(10):[1509-16 pp.]. Available from: http://onlinelibrary.wiley.com/o/cochrane/clcentral/articles/691/CN-00880691/frame.html.
46. Inoue Y, Saito T, Ogawa K, Nishio Y, Kosugi S, Suzuki Y, et al. Drug interactions between micafungin at high doses and cyclosporine A in febrile neutropenia patients after allogeneic hematopoietic stem cell transplantation. International journal of clinical pharmacology and therapeutics [Internet]. 2012; 50(11):[831-7 pp.]. Available from: http://onlinelibrary.wiley.com/o/cochrane/clcentral/articles/056/CN-00879056/frame.html.
47. Jeong SH, Kim DY, Jang JH, Mun YC, Choi CW, Kim SH, et al. Efficacy and safety of micafungin versus intravenous itraconazole as empirical antifungal therapy for febrile neutropenic patients with hematological malignancies: a randomized, controlled, prospective, multicenter study. Annals of hematology [Internet]. 2016; 95(2):[337-44 pp.]. Available from: http://onlinelibrary.wiley.com/o/cochrane/clcentral/articles/917/CN-01133917/frame.html.
48. Kale-Pradhan PB, Morgan G, Wilhelm SM, Johnson LB. Comparative efficacy of echinocandins and nonechinocandins for the treatment of Candida parapsilosis infections: A meta-analysis. Pharmacotherapy. 2010;30(12):1207-13.
49. Keirns J, Sawamoto T, Holum M, Buell D, Wisemandle W, Alak A. Steady-state pharmacokinetics of micafungin and voriconazole after separate and concomitant dosing in healthy adults. Antimicrobial agents and chemotherapy [Internet]. 2007; 51(2):[787-90 pp.]. Available from: http://onlinelibrary.wiley.com/o/cochrane/clcentral/articles/216/CN-00586216/frame.html.
50. Khawcharoenporn T, Apisarnthanarak A, Mundy LM. Treatment of cryptococcosis in the setting of HIV coinfection. Expert Review of Anti-Infective Therapy. 2007;5(6):1019-30.
51. Knitsch W, Vincent JL, Utzolino S, François B, Dinya T, Dimopoulos G, et al. A randomized, placebo-controlled trial of preemptive antifungal therapy for the

prevention of invasive candidiasis following gastrointestinal surgery for intra-abdominal infections. Clinical infectious diseases : an official publication of the Infectious Diseases Society of America [Internet]. 2015; 61(11):[1671-8 pp.]. Available from: http://onlinelibrary.wiley.com/o/cochrane/clcentral/articles/333/CN-01170333/frame.html.
52. Kobayashi C, Hanadate T, Niwa T, Yoshiyasu T, So M, Matsui K. Safety and Effectiveness of Micafungin in Japanese Pediatric Patients: Results of a Postmarketing Surveillance Study. Journal of pediatric hematology/oncology [Internet]. 2015; 37(5):[e285-e91 pp.]. Available from: http://onlinelibrary.wiley.com/o/cochrane/clcentral/articles/509/CN-01134509/frame.html.
53. Kobayashi R, Suzuki D, Sano H, Kishimoto K, Yasuda K, Kobayashi K. Effect of meropenem with or without immunoglobulin as second-line therapy for pediatric febrile neutropenia. Pediatrics international [Internet]. 2014; 56(4):[526-9 pp.]. Available from: http://onlinelibrary.wiley.com/o/cochrane/clcentral/articles/384/CN-01068384/frame.html.
54. Kohno S, Izumikawa K, Ogawa K, Kurashima A, Okimoto N, Amitani R, et al. Intravenous micafungin versus voriconazole for chronic pulmonary aspergillosis: a multicenter trial in Japan. The Journal of infection [Internet]. 2010; 61(5):[410-8 pp.]. Available from: http://onlinelibrary.wiley.com/o/cochrane/clcentral/articles/616/CN-00789616/frame.html.
55. Kohno S, Izumikawa K, Yoshida M, Takesue Y, Oka S, Kamei K, et al. A double-blind comparative study of the safety and efficacy of caspofungin versus micafungin in the treatment of candidiasis and aspergillosis. European journal of clinical microbiology & infectious diseases : official publication of the European Society of Clinical Microbiology [Internet]. 2013; 32(3):[387-97 pp.]. Available from: http://onlinelibrary.wiley.com/o/cochrane/clcentral/articles/619/CN-00878619/frame.html.
56. Kreutzkamp B. Candidemia and invasive candidosis: Effective first-line treatment with micafungin. Arzneimitteltherapie. 2008;26(2):75.
57. Krishna G, Vickery D, Ma L, Yu X, Noren C, Power E, et al. Lack of pharmacokinetic drug interaction between oral posaconazole and caspofungin or micafungin. Journal of clinical pharmacology [Internet]. 2011; 51(1):[84-92 pp.]. Available from:

http://onlinelibrary.wiley.com/o/cochrane/clcentral/articles/947/CN-00777947/frame.html.
58. Kuse ER, Chetchotisakd P, da Cunha CA, Ruhnke M, Barrios C, Raghunadharao D, et al. Micafungin versus liposomal amphotericin B for candidaemia and invasive candidosis: a phase III randomised double-blind trial. Lancet. 2007;369(9572):1519-27. Epub 2007/05/08. doi: 10.1016/s0140-6736(07)60605-9. PubMed PMID: 17482982.
59. Lewis RE. Pharmacotherapy of Candida bloodstream infections: New treatment options, new era. Expert Opinion on Pharmacotherapy. 2002;3(8):1039-57.
60. Maede Y, Ibara S, Nagasaki H, Inoue T, Tokuhisa T, Torikai M. Micafungin versus fluconazole for prophylaxis against fungal infections in premature infants. Pediatrics International [Internet]. 2013; 55(6):[727-30 pp.]. Available from: http://onlinelibrary.wiley.com/o/cochrane/clcentral/articles/331/CN-00973331/frame.html.
61. Maertens JA, Frère P, Lass-Flörl C, Heinz W, Cornely OA. Primary antifungal prophylaxis in leukaemia patients. European Journal of Cancer, Supplement. 2007;5(2):43-8.
62. Marr K. Combination antifungal therapy: where are we now, and where are we going? Oncology (Williston Park, NY). 2004;18(13 Suppl 7):24-9.
63. Matsumoto Y, Murat D, Kojima T, Shimazaki J, Tsubota K. The comparison of solitary topical micafungin or fluconazole application in the treatment of Candida fungal keratitis. British journal of ophthalmology [Internet]. 2011; 95(10):[1406-9 pp.]. Available from: http://onlinelibrary.wiley.com/o/cochrane/clcentral/articles/120/CN-00895120/frame.html.
64. McCoy D, DePestel DD, Carver PL. Primary antifungal prophylaxis in adult hematopoietic stem cell transplant recipients: Current therapeutic concepts. Pharmacotherapy. 2009;29(11):1306-25.
65. Michael AP. Head-to-head comparison of micafungin versus caspofungin in the treatment of invasive candidiasis. Current fungal infection reports [Internet]. 2009; 3(3):[127-8 pp.]. Available from: http://onlinelibrary.wiley.com/o/cochrane/clcentral/articles/446/CN-00889446/frame.html.
66. Miglietta F, Vella A, Faneschi ML, Lobreglio G, Rizzo A, Palumbo C, et al. Geotrichum capitatum septicaemia in a haematological patient after acute myeloid leukaemia relapse: Identification using MALDI-TOF mass spectrometry and review of the literature. Infezioni in Medicina. 2015;23(2):161-7.

67. Mills EJ, Perri D, Cooper C, Nachega JB, Wu P, Tleyjeh I, et al. Antifungal treatment for invasive Candida infections: A mixed treatment comparison meta-analysis. Annals of Clinical Microbiology and Antimicrobials. 2009;8.
68. Moen MD, Lyseng-Williamson KA, Scott LJ. Liposomal amphotericin B: A review of its use as empirical therapy in febrile neutropenia and in the treatment of invasive fungal infections. Drugs. 2009;69(3):361-92.
69. Muñoz P, Guinea J, Narbona MT, Bouza E. Treatment of invasive fungal infections in immunocompromised and transplant patients: AmBiLoad Trial and other new data. International Journal of Antimicrobial Agents. 2008;32(SUPPL. 2):S125-S31.
70. Munoz P, Valerio M, Palomo J, Giannella M, Yanez JF, Desco M, et al. Targeted antifungal prophylaxis in heart transplant recipients. Transplantation [Internet]. 2013; 96(7):[664-9 pp.]. Available from: http://onlinelibrary.wiley.com/o/cochrane/clcentral/articles/204/CN-00910204/frame.html.
71. Murali S, Langston A. Advances in antifungal prophylaxis and empiric therapy in patients with hematologic malignancies: Review article. Transplant Infectious Disease. 2009;11(6):480-90.
72. Nct. Randomized, open label, non-inferiority study of micafungin versus standard care for the prevention of invasive fungal disease in high risk liver transplant recipients. clinicaltrialsgov/ct2/show/NCT01058174 [Internet]. 2010. Available from: http://onlinelibrary.wiley.com/o/cochrane/clcentral/articles/196/CN-00766196/frame.html.
73. Neoh CF, Liew D, Slavin MA, Marriott D, Chen SC, Morrissey O, et al. Economic evaluation of micafungin versus caspofungin for the treatment of candidaemia and invasive candidiasis. Internal medicine journal [Internet]. 2013; 43(6):[668-77 pp.]. Available from: http://onlinelibrary.wiley.com/o/cochrane/clcentral/articles/045/CN-01124045/frame.html.
74. Oyake T, Kowata S, Murai K, Ito S, Akagi T, Kubo K, et al. Comparison of micafungin and voriconazole as empirical antifungal therapies in febrile neutropenic patients with hematological disorders: A randomized controlled trial. European journal of haematology [Internet]. 2016; 96(6):[602-9 pp.]. Available from: http://onlinelibrary.wiley.com/o/cochrane/clcentral/articles/775/CN-01159775/frame.html.
75. Pappas PG, Rotstein CMF, Betts RF, Nucci M, Talwar D, De Waele JJ, et al. Micafungin versus caspofungin for treatment of candidemia and other forms of invasive candidiasis. Clinical Infectious Diseases. 2007;45(7):883-93.

76. Park HJ, Park M, Han M, Nam BH, Koh KN, Im HJ, et al. Efficacy and safety of micafungin for the prophylaxis of invasive fungal infection during neutropenia in children and adolescents undergoing allogeneic hematopoietic SCT. Bone marrow transplantation [Internet]. 2014; 49(9):[1212-6 pp.]. Available from: http://onlinelibrary.wiley.com/o/cochrane/clcentral/articles/810/CN-01001810/frame.html.
77. Park S, Kim K, Jang JH, Kim SJ, Kim WS, Chung DR, et al. Randomized trial of micafungin versus fluconazole as prophylaxis against invasive fungal infections in hematopoietic stem cell transplant recipients. Journal of Infection. 2016.
78. Perfect JR. Management of invasive mycoses in hematology patients: current approaches. Oncology (Williston Park, NY). 2004;18(13 Suppl 7):5-14.
79. Pettengell K, Mynhardt J, Kluyts T, Lau W, Facklam D, Buell D. Successful treatment of oesophageal candidiasis by micafungin: a novel systemic antifungal agent. Alimentary pharmacology & therapeutics [Internet]. 2004; 20(4):[475-81 pp.]. Available from: http://onlinelibrary.wiley.com/o/cochrane/clcentral/articles/632/CN-00490632/frame.html.
80. Queiroz-Telles F, Berezin E, Leverger G, Freire A, Vyver A, Chotpitayasunondh T, et al. Micafungin versus liposomal amphotericin B for pediatric patients with invasive candidiasis: substudy of a randomized double-blind trial. The Pediatric infectious disease journal [Internet]. 2008; 27(9):[820-6 pp.]. Available from: http://onlinelibrary.wiley.com/o/cochrane/clcentral/articles/265/CN-00650265/frame.html.
81. Robenshtok E, Gafter-Gvili A, Goldberg E, Weinberger M, Yeshurun M, Leibovici L, et al. Antifungal prophylaxis in cancer patients after chemotherapy or hematopoietic stem-cell transplantation: Systematic review and meta-analysis. Journal of Clinical Oncology. 2007;25(34):5471-89.
82. Rueping MJGT, Vehreschild JJ, Cornely OA. Invasive candidiasis and candidemia: From current opinions to future perspectives. Expert Opinion on Investigational Drugs. 2009;18(6):735-48.
83. Saliba F, Pascher A, Cointault O, Laterre PF, Cervera C, Waele JJ, et al. Randomized trial of micafungin for the prevention of invasive fungal infection in high-risk liver transplant recipients. Clinical infectious diseases : an official publication of the Infectious Diseases Society of America [Internet]. 2015; 60(7):[997-1006 pp.]. Available from: http://onlinelibrary.wiley.com/o/cochrane/clcentral/articles/692/CN-01070692/frame.html.

84. Sawada A, Sakata N, Higuchi B, Takeshita Y, Ishihara T, Sakata A, et al. [Comparison of micafungin and fosfluconazole as prophylaxis for invasive fungal infection during neutropenia in children undergoing chemotherapy and hematopoietic stem cell transplantation]. [Rinsho ketsueki] The Japanese journal of clinical hematology [Internet]. 2009; 50(12):[1692-9 pp.]. Available from: http://onlinelibrary.wiley.com/o/cochrane/clcentral/articles/065/CN-00734065/frame.html.
85. Shang W, Feng G, Sun R, Wang X, Liu W, Zhang S, et al. Comparison of micafungin and voriconazole in the treatment of invasive fungal infections in kidney transplant recipients. Journal of clinical pharmacy and therapeutics [Internet]. 2012; 37(6):[652-6 pp.]. Available from: http://onlinelibrary.wiley.com/o/cochrane/clcentral/articles/901/CN-00968901/frame.html.
86. Shorr AF, Wu C, Kothari S. Outcomes with micafungin in patients with candidaemia or invasive candidiasis due to Candida glabrata and Candida krusei. The Journal of antimicrobial chemotherapy [Internet]. 2011; 66(2):[375-80 pp.]. Available from: http://onlinelibrary.wiley.com/o/cochrane/clcentral/articles/213/CN-00802213/frame.html.
87. Tamura K. Clinical guidelines for the management of neutropenic patients with unexplained fever in Japan: Validation by the Japan Febrile Neutropenia Study Group. International journal of antimicrobial agents [Internet]. 2005; 26(Suppl. 2):[S123-s7 pp.]. Available from: http://onlinelibrary.wiley.com/o/cochrane/clcentral/articles/637/CN-00557637/frame.html.
88. Tamura K, Drew R. Antifungal prophylaxis in adult hematopoietic stem cell transplant recipients. Drugs of today (Barcelona, Spain : 1998) [Internet]. 2008; 44(7):[515-30 pp.]. Available from: http://onlinelibrary.wiley.com/o/cochrane/clcentral/articles/892/CN-00914892/frame.html.
89. Temesgen Z, Barreto J, Vento S. Micafungin - The newest echinocandin. Drugs of today (Barcelona, Spain : 1998) [Internet]. 2009; 45(6):[469-78 pp.]. Available from: http://onlinelibrary.wiley.com/o/cochrane/clcentral/articles/876/CN-00958876/frame.html.
90. Timsit JF, Azoulay E, Cornet M, Gangneux JP, Jullien V, Vésin A, et al. EMPIRICUS micafungin versus placebo during nosocomial sepsis in Candida multi-colonized ICU patients with multiple organ failures: study protocol for a randomized controlled trial. Trials [Internet]. 2013; 14:[399 p.]. Available from:

http://onlinelibrary.wiley.com/o/cochrane/clcentral/articles/469/CN-01015469/frame.html.
91. Undre N, Stevenson P, Baraldi E. Pharmacokinetics of micafungin in HIV positive patients with confirmed esophageal candidiasis. European journal of drug metabolism and pharmacokinetics [Internet]. 2012; 37(1):[31-8 pp.]. Available from: http://onlinelibrary.wiley.com/o/cochrane/clcentral/articles/907/CN-00971907/frame.html.
92. Van Burik JAH, Ratanatharathorn V, Stepan DE, Miller CB, Lipton JH, Vesole DH, et al. Micafungin versus fluconazole for prophylaxis against invasive fungal infections during neutropenia in patients undergoing hematopoietic stem cell transplantation. Clinical Infectious Diseases. 2004;39(10):1407-16.
93. Wan L, Zhang Y, Lai Y, Jiang M, Song Y, Zhou J, et al. Effect of Granulocyte-Macrophage Colony-Stimulating Factor on Prevention and Treatment of Invasive Fungal Disease in Recipients of Allogeneic Stem-Cell Transplantation: A Prospective Multicenter Randomized Phase IV Trial. Journal of clinical oncology : official journal of the American Society of Clinical Oncology [Internet]. 2015; 33(34):[3999-4006 pp.]. Available from: http://onlinelibrary.wiley.com/o/cochrane/clcentral/articles/337/CN-01128337/frame.html.
94. Wet N, Llanos-Cuentas A, Suleiman J, Baraldi E, Krantz EF, Della Negra M, et al. A randomized, double-blind, parallel-group, dose-response study of micafungin compared with fluconazole for the treatment of esophageal candidiasis in HIV-positive patients. Clinical infectious diseases : an official publication of the Infectious Diseases Society of America [Internet]. 2004; 39(6):[842-9 pp.]. Available from: http://onlinelibrary.wiley.com/o/cochrane/clcentral/articles/636/CN-00527636/frame.html.
95. Wet NT, Bester AJ, Viljoen JJ, Filho F, Suleiman JM, Ticona E, et al. A randomized, double blind, comparative trial of micafungin (FK463) vs. fluconazole for the treatment of oesophageal candidiasis. Alimentary pharmacology & therapeutics [Internet]. 2005; 21(7):[899-907 pp.]. Available from: http://onlinelibrary.wiley.com/o/cochrane/clcentral/articles/544/CN-00512544/frame.html.
96. Wheat LJ. Combination therapy for aspergillosis: Is it needed, and which combination? Journal of Infectious Diseases. 2003;187(12):1831-3.
97. Wingard JR. New approaches to invasive fungal infections in acute leukemia and hematopoietic stem cell transplant patients. Best Practice and Research in Clinical Haematology. 2007;20(1):99-107.

98. Wong-Beringer A, Kriengkauykiat J. Systemic Antifungal Therapy: New Options, New Challenges. Pharmacotherapy. 2003;23(11):1441-62.
99. Yamaguchi H, Enomoto S, Kaku M, Sakamaki H, Tanaka K, Yoshida M. An open randomized parallel-comparison study of itraconazole oral solution versus itraconazole capsules in treatment of patients with oropharyngeal candidiasis. [Japanese]. Japanese Journal of Chemotherapy [Internet]. 2006; 54(Suppl. 1):[18-31 pp.]. Available from: http://onlinelibrary.wiley.com/o/cochrane/clcentral/articles/751/CN-00612751/frame.html.
100. Zhang AY, Camp WL, Elewski BE. Advances in Topical and Systemic Antifungals. Dermatologic Clinics. 2007;25(2):165-83.
101. Mahmoud A.M., Shalaby L.M. Micafungin as a prophylactic antifungal during induction phase of chemotherapy for pediatric acute lymphoblastic leukemia. M.B, B.Ch, Cairo University. MSc degree. 2016.
