# Supplementary material for: Efficacy and safety of micafungin versus extensive azoles in the prevention and treatment of invasive fungal infections for neutropenia patients with hematological malignancies: A meta-analysis of randomized controlled trials
Source: PLoS One. 2017 Jul 12;12(7):e0180050. doi: 10.1371/journal.pone.0180050 (PMC5507498; doi:10.1371/journal.pone.0180050)
Supplement: S3 Table — (PDF) [file pone.0180050.s003.pdf]

Table 3. Quality asscessment

	Random sequence generation (selection bias)	Allocation concealment (selection bias)	Blinding of participants/personnel All outcomes (performance bias)	Blinding of outcome assessment All outcomes (detection bias)	Incomplete outcome data All outcomes (attrition bias)	Selective reporting (reporting bias)	Other bias
Burik 2004	The randomization schedule was generated by the Research Data Operations Department of Fujisawa Healthcare."	Pharmacists involved in the investigation randomized the patients via an interactive central telephone service.	To maintain the blind, all patients received the daily infusion intravenously, in a volume of 200 mL over 1 h, from an infusion set covered by an opaque bag." Hospital staff at study centers and all nonpharmacist study staff involved in the investigation were blinded to the randomized drug	Breakthrough invasive fungal infections that were determined by investigators to be proven or probable were reviewed in a blinded manner by means of predetermined protocol criteria...All data were reviewed and analyzed by the MSG Central Biostatistics Unit (University of Alabama, Birmingham). "	Modified intention-to-treat efficacy analysis discontinued 6.9%;5.2%	Important outcomes were congruent with trial's protocol.	charact isic fique p>0.05

Hiemenz 2005	The authors did not describe the method of avoiding selection bias.	The authors did not describe the method of avoiding selection bias.	Micafungin and saline were administered as 100-ml intravenous infusions over 1 h in a blind manner." Comment:Probably done	The authors did not mention the blinding of outcome assessment, but with objective outcome	No patients dropping out from both Micagungin and fluconazole treatment groups, but not mentioned about analysis model	The main outcomes of prophylaxis against fungal infections were reported.	charact isic fique p>0.05
Hiramatsu 2008	The autor did not clearly descride the method of random sequence generation.	Patients who provided informed consent were randomly assigned to receive either micafungin or fluconazole using a 1:1 schedule."	The study was a prospective, randomized, open-labeled comparative trial...	The authors did not mention the blinding of outcome assessment but with objective outcome Comment: Altough the study is an open-label trial, the outcome is not likely to be influenced by lack of blinding.	Modified intention-to-treat efficacy analysis discountined 2%;2%	Important outcomes were congruent with trial's protocol.	charact isic fique p>0.05
Sawada 2009	not mentioned	randomly assigned to receive either micafungin or	randomized, open-labeled comparative trial...	randomized, open-labeled comparative trial... Comment: Altough the study	Modified intention-to-treat efficacy analysis	Important outcomes were	charact isic fique

		fluconazole using a 1:1 schedule		is an open-label trial, the outcome is not likely to be influenced by lack of blinding.	discountined 1%;1%"	congruent with trial's protocol.	p>0.05
Huang 2012	...by block randomization using randomization codes generated by SAS PROC Plan."	...by block randomization using randomization codes generated by SAS PROC Plan.	Randomized, open-label trial…	Open-label trial. Comment: Altough the study is an open-label trial, the outcome is not likely to be influenced by lack of blinding.	intention-to-treat discontinued 14.7%;32.6% high drop out rate	Reporting bias is probably not seen.	charact isic fique p>0.05
Oyake 2015	Centralized website (SAS statistical software version 8.02) according to two variables: risk (high or low) and systemic antifungal prophylaxis (yes or no)." Comment: Probably done.	Centralized website (SAS statistical software version 8.02) according to two variables: risk (high or low) and systemic antifungal prophylaxis (yes or no)." Comment: Probably done.	" Randomized, open-label trial…	Efficacy and safety assessments were performed by an independent data monitoring committee. Comment: Probably done.	Modified intention-to-treat population discontinued f/u: 2%;10%	The main outcomes of prophylaxis against fungal infections were reported.	charact isic fique p>0.05

Jeong 2016	...they were randomly assigned, in a 1:1 ratio by block randomization...	Subjects were automatically assigned by a pre-made random code with a Web-based electronic system.	The author does not mention the method of blinding	"open-label". Comment: Altough the study is an open-label trial, the outcome is not likely to be influenced by lack of blinding.	intention-to-treat discontinued 26%;22%	The main outcomes of prophylaxis against fungal infections were reported.	charact isic fique p>0.05
Park 2016	The author did not mention.	A total of 257 patients were randomly assigned to one of the two treatment arms. Comment: Probably not done.	The author does not mention the method of blinding. Comparative drug was oral fluzonazole	"open-label". Comment: Altough the study is an open-label trial, the outcome is not likely to be influenced by lack of blinding.	intention-to-treat discontinued 6.1%; 9.4%	not mentioned about adverse events	charact isic fique p>0.05
Mahmoud 2016	The author did not mention.	The author did not mention.	The author does not mention the method of blinding	"open-label". Comment: Altough the study is an open-label trial, the outcome is not likely to be influenced by lack of blinding.	Modified intention-to-treat efficacy analysis, no discontinued	not mentioned about adverse events	charact isic fique p>0.05
