# Supplementary material for: Efficacy and safety of micafungin versus extensive azoles in the prevention and treatment of invasive fungal infections for neutropenia patients with hematological malignancies: A meta-analysis of randomized controlled trials
Source: PLoS One. 2017 Jul 12;12(7):e0180050. doi: 10.1371/journal.pone.0180050 (PMC5507498; doi:10.1371/journal.pone.0180050)
Supplement: S4 Table — (PDF) [file pone.0180050.s004.pdf]

S4 Table A. Data of Included Randomized Trials

Study	Treatment Success rate				Fungal infection, overall				IFI, proven				IFI, probably				IFI, possible				Rotation of anti-fungal agent				Discontinued prematurely			
	Event (MCFG)	Sample (MCFG)	Event (Azoles)	Sample (Azoles)	Event (MCFG)	Sample (MCFG)	Event (Azoles)	Sample (Azoles)	Event (MCFG)	Sample (MCFG)	Event (Azoles)	Sample (Azoles)	Event (MCFG)	Sample (MCFG)	Event (Azoles)	Sample (Azoles)	Event (MCFG)	Sample (MCFG)	Event (Azoles)	Sample (Azoles)	Event (MCFG)	Sample (MCFG)	Event (Azoles)	Sample (Azoles)	Event (MCFG)	Sample (MCFG)	Event (Azoles)	Sample (Azoles)
Burik et al. 2004 (14)	340	425	336	457	71	425	109	457	6	425	8	457	1	425	3	457	64	425	98	457	253	425	298	457	18	425	33	457
Hiemenz et al. 2005 (29)	48	62	7	12	14	62	5	12	2	62	2	12	1	62	0	12	11	62	3	12	14	62	5	12	2	62	1	12
Hiramatsu et al. 2008 (30)	47	50	44	50	3	50	7	50	1	50	1	50	0	50	0	50	2	50	6	50	2	50	6	50	0	50	0	50
Sawada et al. 2009 (31)	48	54	31	53	3	54	3	53	0	54	0	53	3	54	3	53	0	54	0	53	NA	NA	NA	NA	NA	NA	NA	NA
Huang et al. 2012 (32)	125	136	109	147	14	136	13	147	1	136	0	147	5	136	2	147	8	136	11	147	34	136	35	147	10	136	35	147
Oyake et al. 2015 (9)	48	49	45	45	2	49	6	45	0	49	0	45	1	49	2	45	1	49	4	45	12	49	28	45	12	49	28	45
Jeong et al. 2016 (8)	47	73	43	75	23	73	26	75	1	73	2	75	2	73	3	75	20	73	21	75	23	73	26	75	23	73	26	75
Park et al. 2016 (34)	153	165	78	85	19	165	9	85	2	165	3	85	11	165	5	85	6	165	1	85	13	165	8	85	4	165	5	85
Mahmoud et al. 2016 (7)	33	35	27	35	2	35	8	35	0	35	0	35	2	35	6	35	0	35	2	35	2	35	22	35	NA	NA	NA	NA

S4 Table A. Data of Included Randomized Trials (continued)

Study	All-caused Mortality				Adverse events, overall				Adverse Events, mild				Adverse Events, severe				AE, allergic reaction				AE, hepatic impairment				AE, neurologic symptoms			
	Event (MCFG)	Sample (MCFG)	Event (Azoles)	Sample (Azoles)	Event (MCFG)	Sample (MCFG)	Event (Azoles)	Sample (Azoles)	Event (MCFG)	Sample (MCFG)	Event (Azoles)	Sample (Azoles)	Event (MCFG)	Sample (MCFG)	Event (Azoles)	Sample (Azoles)	Event (MCFG)	Sample (MCFG)	Event (Azoles)	Sample (Azoles)	Event (MCFG)	Sample (MCFG)	Event (Azoles)	Sample (Azoles)	Event (MCFG)	Sample (MCFG)	Event (Azoles)	Sample (Azoles)
Burik et al. 2004 (14)	18	425	26	457	68	425	87	457	64	425	77	457	4	425	10	457	8	425	6	457	22	425	31	457	NA	NA	NA	NA
Hiemenz et al. 2005 (29)	4	62	1	12	16	62	6	12	14	62	4	12	2	62	2	12	2	62	2	12	NA	NA	NA	NA	4	62	4	12
Hiramatsu et al. 2008 (30)	4	50	2	50	11	50	9	50	7	50	8	50	4	50	1	50	0	50	0	50	5	50	5	50	NA	NA	NA	NA
Sawada et al. 2009 (31)	NA	NA	NA	NA	15	54	20	53	13	54	10	53	2	54	10	53	NA	NA	NA	NA	NA	NA	NA	NA	NA	NA	NA	NA
Huang et al. 2012 (32)	0	136	1	147	11	136	39	147	11	136	39	147	0	136	0	147	0	136	0	147	9	136	7	147	NA	NA	NA	NA
Oyake et al. 2015 (9)	1	49	0	45	10	49	28	45	5	49	14	45	5	49	14	45	2	49	9	45	3	49	6	45	0	49	3	45
Jeong et al. 2016 (8)	6	73	8	75	51	73	44	75	40	73	27	75	11	73	17	75	9	73	5	75	15	73	30	75	NA	NA	NA	NA
Park et al. 2016 (34)	15	165	11	85	3	165	2	85	3	165	2	85	0	165	0	85	NA	NA	NA	NA	NA	NA	NA	NA	NA	NA	NA	NA
Mahmoud et al. 2016 (7)	0	35	4	35	NA	NA	NA	NA	NA	NA	NA	NA	NA	NA	NA	NA	NA	NA	NA	NA	NA	NA	NA	NA	NA	NA	NA	NA

S4 Table B. Moderators for meta-regression of Included Randomized Trials

Study	Underlying Therapy	Quality (Risk of bias)	Mean Age (year)	Allogenic SCT (%)	Neutropenia duration (D)	Leukemia (%)
Burik et al. 2004 (14)	Transplant	7	42.53	53.90%	13	29.0%
Hiemenz et al. 2005 (29)	Transplant	4	43.24	43.20%	NA	NA
Hiramatsu et al. 2008 (30)	Transplant	3	46.90	52.00%	13.7	19.0%
Sawada et al. 2009 (31)	Transplant & Chemotherapy	5	6.01	14.00%	NA	27.0%
Huang et al. 2012 (32)	Transplant	4	32.72	80.20%	15	49.0%
Oyake et al. 2015 (9)	Chemotherapy	6	53.00	NA	19	77.0%
Jeong et al. 2016 (8)	Chemotherapy	6	49.00	NA	16	86.0%
Park et al. 2016 (34)	Transplant	3	46.66	56.00%	NA	41.0%
Mahmoud et al. 2016 (7)	Chemotherapy	3	7.35	NA	NA	100.0%
