# Supplementary figures and images for: Efficacy and safety of micafungin versus extensive azoles in the prevention and treatment of invasive fungal infections for neutropenia patients with hematological malignancies: A meta-analysis of randomized controlled trials
Source: PLoS One. 2017 Jul 12;12(7):e0180050. doi: 10.1371/journal.pone.0180050 (PMC5507498; doi:10.1371/journal.pone.0180050)

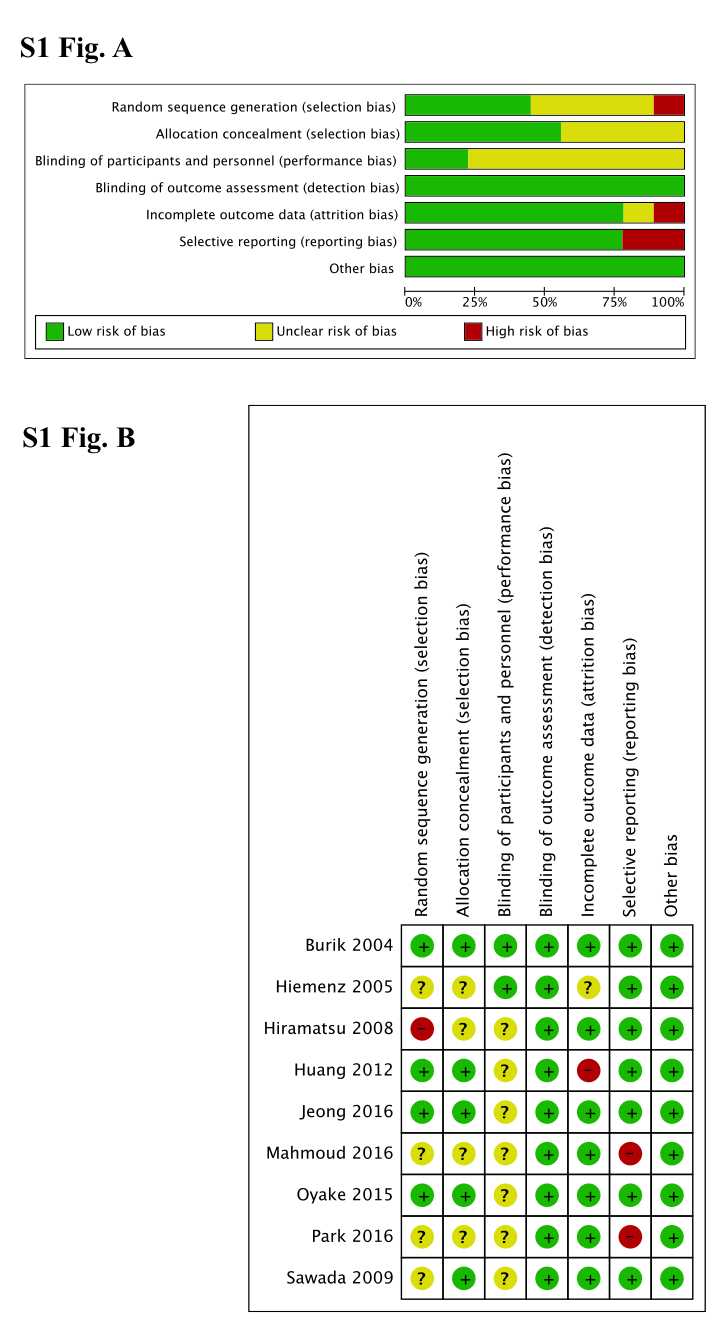

Supplement: S1 Fig — (A) Risk of bias graph. (B) Risk of bias summary. (TIFF) [file pone.0180050.s005.tiff]

A

Treatment Success Rates

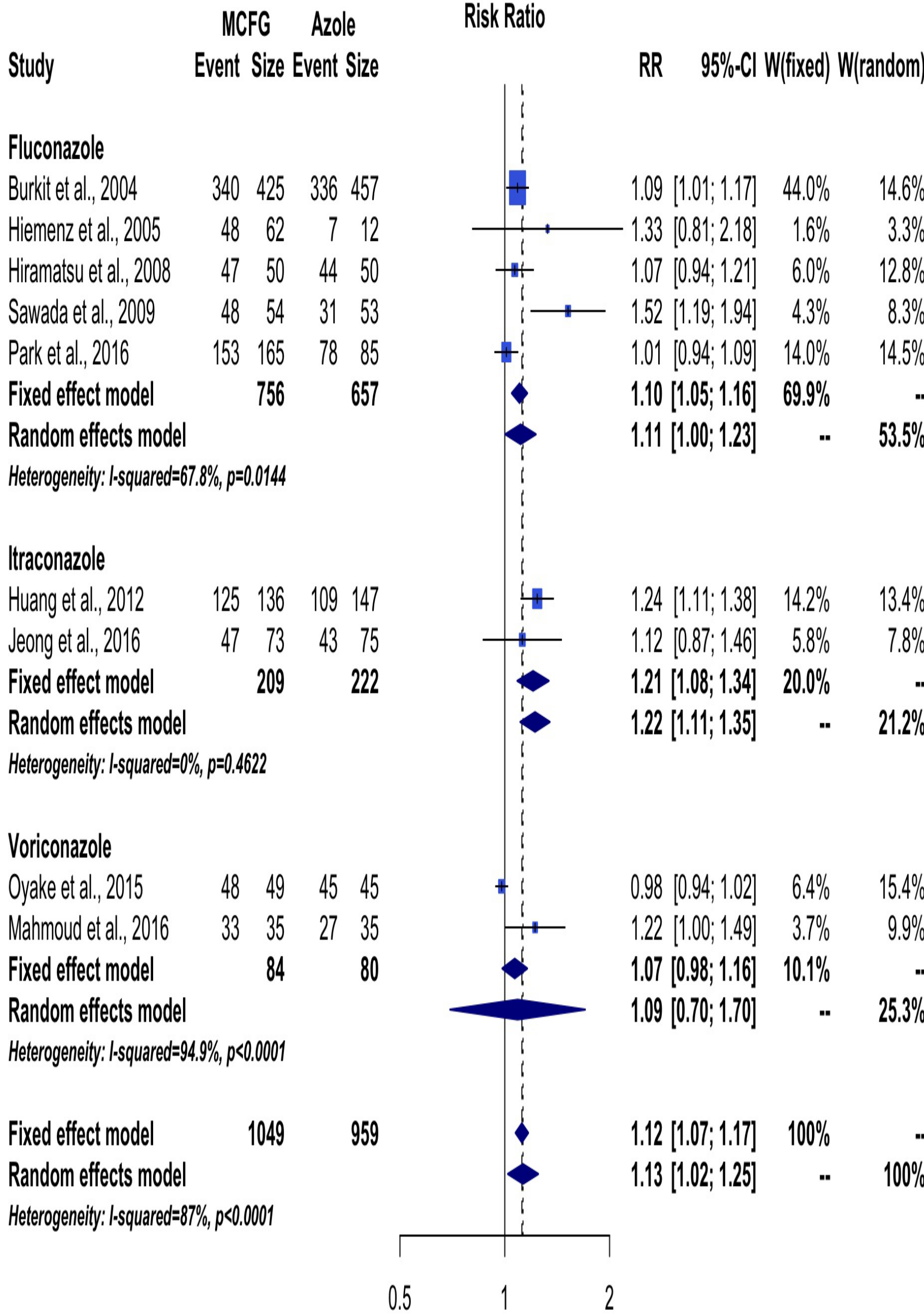

B

Fungal Infection, Overall

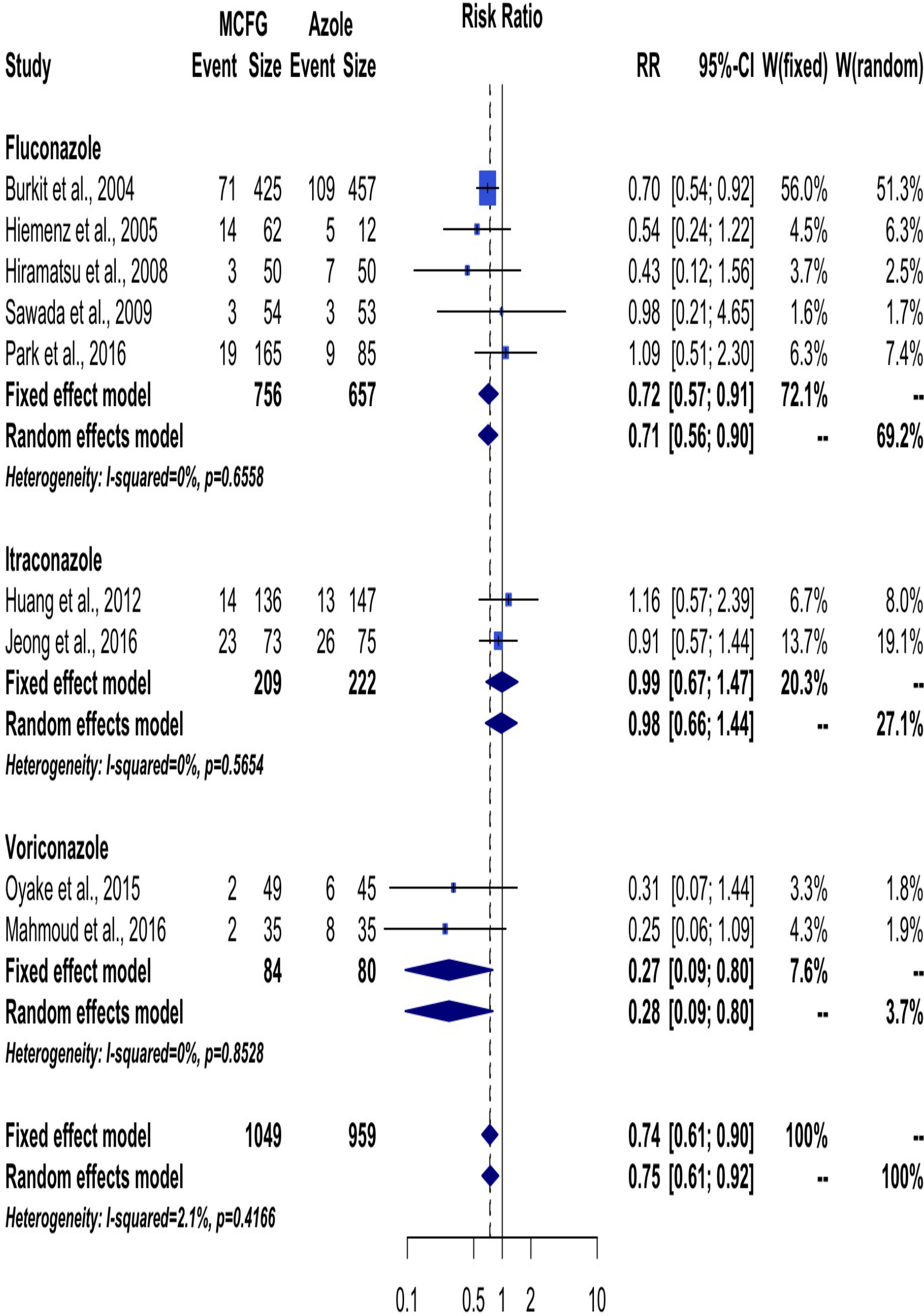

C

Adeverse Events, Ovaerall

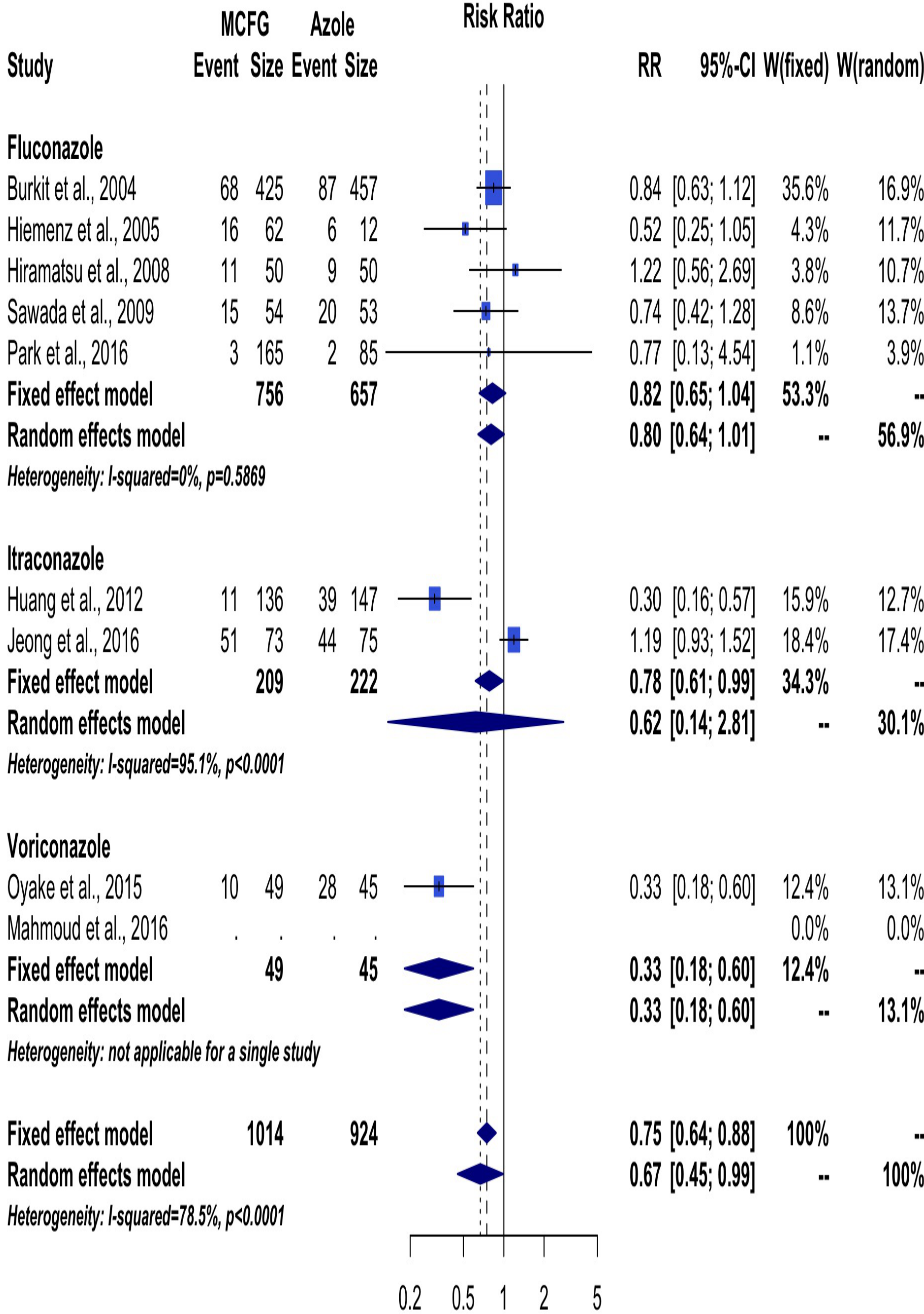

Supplement: S3 Fig — (A) Forest plots of Treatment Success Rates model. (B) Forest plots of Fungal Infection, Overall model. (C) Forest plots of Adverse Events, Overall model. (PDF) [file pone.0180050.s007.pdf]
