# Supplementary material for: Efficacy and safety of micafungin versus extensive azoles in the prevention and treatment of invasive fungal infections for neutropenia patients with hematological malignancies: A meta-analysis of randomized controlled trials
Source: PLoS One. 2017 Jul 12;12(7):e0180050. doi: 10.1371/journal.pone.0180050 (PMC5507498; doi:10.1371/journal.pone.0180050)
Supplement: S2 Fig — (A) Forest plot of rotation of anti-fungal agents model. (B) Forest plot of prematurely discontinued model. (C) Forest plot of All-Caused Mortality model. (D) Forest plot of Adverse Events, Hepatic impairment model. (PDF) [file pone.0180050.s006.pdf]

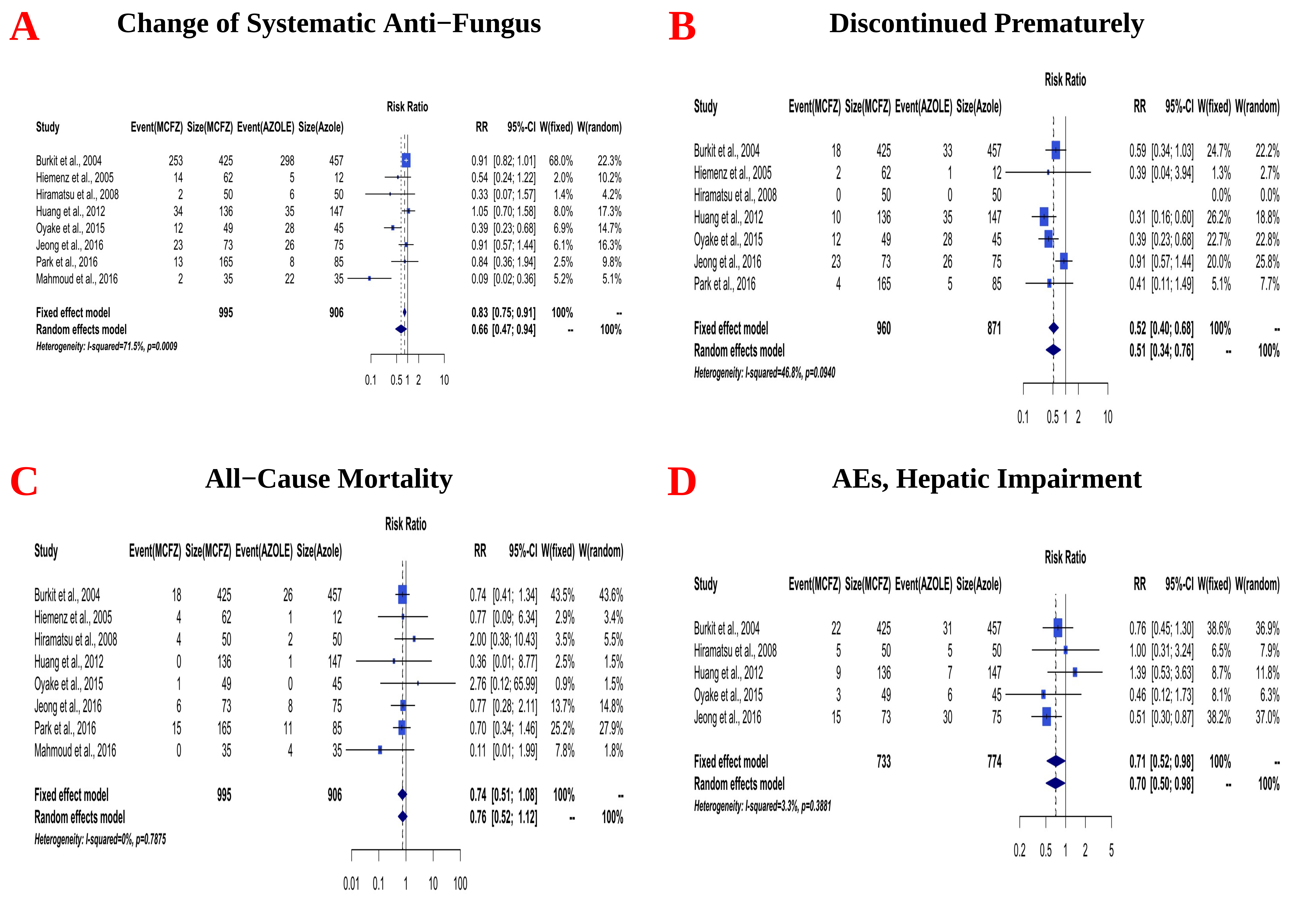
A
Change of Systematic Anti−Fungus
Risk Ratio
Study Event(MCFZ) Size(MCFZ) Event(AZOLE) Size(Azole) RR 95%-CI W(fixed) W(random)
Burkit et al., 2004 253 425 298 457 0.91 [0.82; 1.01] 68.0% 22.3%
Hiemenz et al., 2005 14 62 5 12 0.54 [0.24; 1.22] 2.0% 10.2%
Hiramatsu et al., 2008 2 50 6 50 0.33 [0.07; 1.57] 1.4% 4.2%
Huang et al., 2012 34 136 35 147 1.05 [0.70; 1.58] 8.0% 17.3%
Oyake et al., 2015 12 49 28 45 0.39 [0.23; 0.68] 6.9% 14.7%
Jeong et al., 2016 23 73 26 75 0.91 [0.57; 1.44] 6.1% 16.3%
Park et al., 2016 13 165 8 85 0.84 [0.36; 1.94] 2.5% 9.8%
Mahmoud et al., 2016 2 35 22 35 0.09 [0.02; 0.36] 5.2% 5.1%
Fixed effect model 995 906 0.83 [0.75; 0.91] 100% --
Random effects model 0.66 [0.47; 0.94] -- 100%
Heterogeneity: I-squared=71.5%, p=0.0009
0.1 0.5 1 2 10
B
Discontinued Prematurely
Risk Ratio
Study Event(MCFZ) Size(MCFZ) Event(AZOLE) Size(Azole) RR 95%-CI W(fixed) W(random)
Burkit et al., 2004 18 425 33 457 0.59 [0.34; 1.03] 24.7% 22.2%
Hiemenz et al., 2005 2 62 1 12 0.39 [0.04; 3.94] 1.3% 2.7%
Hiramatsu et al., 2008 0 50 0 50 0.0% 0.0%
Huang et al., 2012 10 136 35 147 0.31 [0.16; 0.60] 26.2% 18.8%
Oyake et al., 2015 12 49 28 45 0.39 [0.23; 0.68] 22.7% 22.8%
Jeong et al., 2016 23 73 26 75 0.91 [0.57; 1.44] 20.0% 25.8%
Park et al., 2016 4 165 5 85 0.41 [0.11; 1.49] 5.1% 7.7%
Fixed effect model 960 871 0.52 [0.40; 0.68] 100% --
Random effects model 0.51 [0.34; 0.76] -- 100%
Heterogeneity: I-squared=46.8%, p=0.0940
0.1 0.5 1 2 10
C
All−Cause Mortality
Risk Ratio
Study Event(MCFZ) Size(MCFZ) Event(AZOLE) Size(Azole) RR 95%-CI W(fixed) W(random)
Burkit et al., 2004 18 425 26 457 0.74 [0.41; 1.34] 43.5% 43.6%
Hiemenz et al., 2005 4 62 1 12 0.77 [0.09; 6.34] 2.9% 3.4%
Hiramatsu et al., 2008 4 50 2 50 2.00 [0.38; 10.43] 3.5% 5.5%
Huang et al., 2012 0 136 1 147 0.36 [0.01; 8.77] 2.5% 1.5%
Oyake et al., 2015 1 49 0 45 2.76 [0.12; 65.99] 0.9% 1.5%
Jeong et al., 2016 6 73 8 75 0.77 [0.28; 2.11] 13.7% 14.8%
Park et al., 2016 15 165 11 85 0.70 [0.34; 1.46] 25.2% 27.9%
Mahmoud et al., 2016 0 35 4 35 0.11 [0.01; 1.99] 7.8% 1.8%
Fixed effect model 995 906 0.74 [0.51; 1.08] 100% --
Random effects model 0.76 [0.52; 1.12] -- 100%
Heterogeneity: I-squared=0%, p=0.7875
0.01 0.1 1 10 100
D
AEs, Hepatic Impairment
Risk Ratio
Study Event(MCFZ) Size(MCFZ) Event(AZOLE) Size(Azole) RR 95%-CI W(fixed) W(random)
Burkit et al., 2004 22 425 31 457 0.76 [0.45; 1.30] 38.6% 36.9%
Hiramatsu et al., 2008 5 50 5 50 1.00 [0.31; 3.24] 6.5% 7.9%
Huang et al., 2012 9 136 7 147 1.39 [0.53; 3.63] 8.7% 11.8%
Oyake et al., 2015 3 49 6 45 0.46 [0.12; 1.73] 8.1% 6.3%
Jeong et al., 2016 15 73 30 75 0.51 [0.30; 0.87] 38.2% 37.0%
Fixed effect model 733 774 0.71 [0.52; 0.98] 100% --
Random effects model 0.70 [0.50; 0.98] -- 100%
Heterogeneity: I-squared=3.3%, p=0.3881
0.2 0.5 1 2 5
