# Supplementary material for: Efficacy and safety of micafungin versus extensive azoles in the prevention and treatment of invasive fungal infections for neutropenia patients with hematological malignancies: A meta-analysis of randomized controlled trials
Source: PLoS One. 2017 Jul 12;12(7):e0180050. doi: 10.1371/journal.pone.0180050 (PMC5507498; doi:10.1371/journal.pone.0180050)
Supplement: S4 Fig — (A) Funnel plot of Treatment Success Rates model. (B) Funnel plot of Fungal Infection, Overall model. (C) Funnel plot of All-Cause Mortality model. (D) Funnel plot of Adverse Events, Overall model. (PDF) [file pone.0180050.s008.pdf]

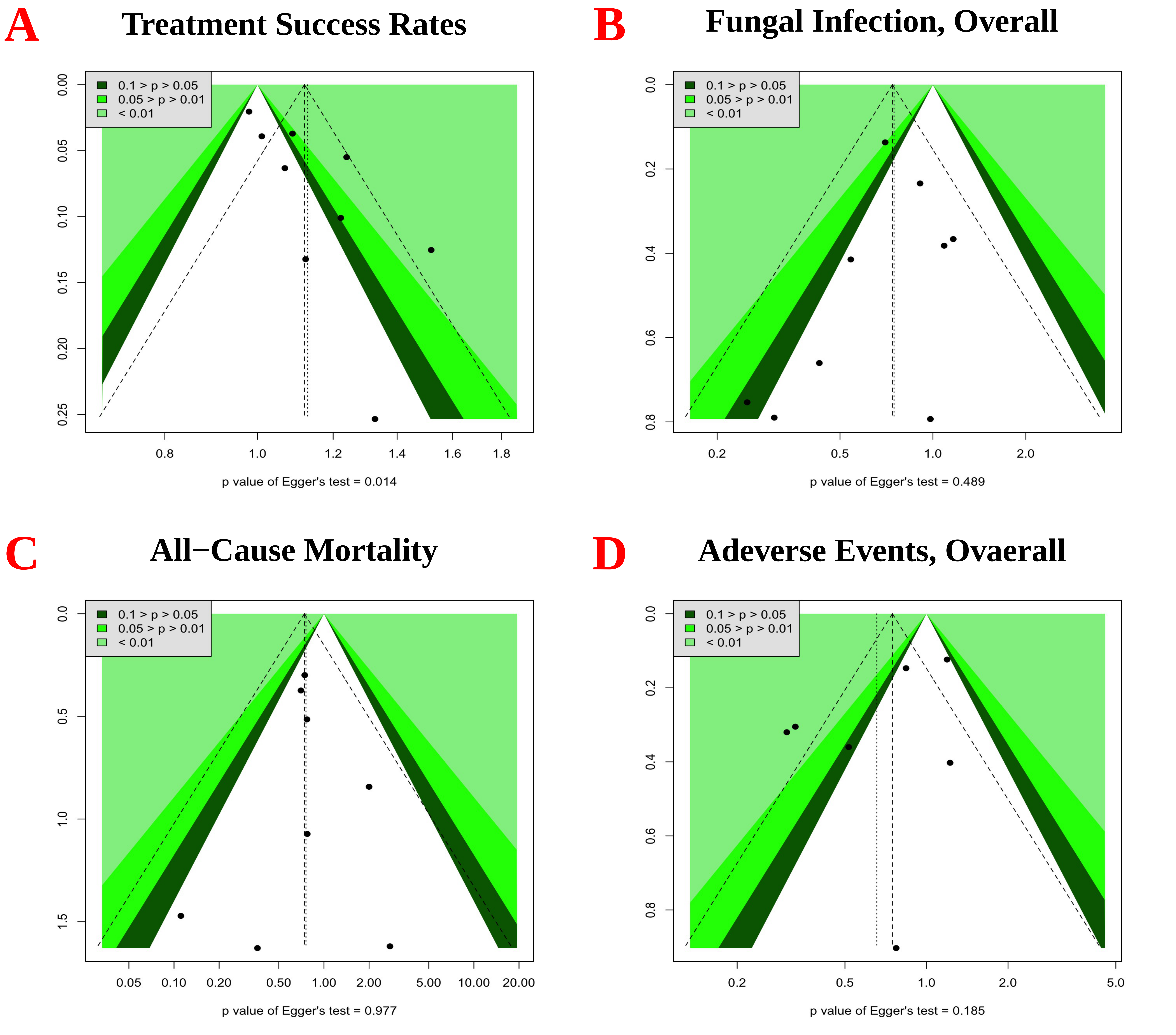
A
Treatment Success Rates
0.1 > p > 0.05
0.05 > p > 0.01
< 0.01
0.00
0.05
0.10
0.15
0.20
0.25
0.8
1.0
1.2
1.4
1.6
1.8
p value of Egger's test = 0.014
B
Fungal Infection, Overall
0.1 > p > 0.05
0.05 > p > 0.01
< 0.01
0.0
0.2
0.4
0.6
0.8
0.2
0.5
1.0
2.0
p value of Egger's test = 0.489
C
All−Cause Mortality
0.1 > p > 0.05
0.05 > p > 0.01
< 0.01
0.0
0.5
1.0
1.5
0.05
0.10
0.20
0.50
1.00
2.00
5.00
10.00
20.00
p value of Egger's test = 0.977
D
Adeverse Events, Ovaerall
0.1 > p > 0.05
0.05 > p > 0.01
< 0.01
0.0
0.2
0.4
0.6
0.8
0.2
0.5
1.0
2.0
5.0
p value of Egger's test = 0.185
